# Deep Learning‐Driven Discovery and Engineering of an Efficient PETase for Depolymerization and Detoxification of PET Microplastics Under Physiological Conditions

**DOI:** 10.1002/advs.77163

**Published:** 2026-08-18

**Authors:** Yuxuan Wang, Shijie He, Yuheng Chang, Sheng Mao, Jianan Canal Li, Binbin Chen, Hongxun Gao, Mingchun Xu, Chenxu Liu, Yajie Wang

**Affiliations:** ^1^ College of Environmental and Resource Sciences Zhejiang University Hangzhou China; ^2^ School of Engineering Westlake University Hangzhou Zhejiang China; ^3^ The Center for Synthetic Biology and Integrated Bioengineering Westlake University Hangzhou Zhejiang China; ^4^ School of Life Science Westlake University Hangzhou Zhejiang China; ^5^ Westlake Laboratory of Life Sciences and Biomedicine Hangzhou Zhejiang Province China; ^6^ Muyuan Laboratory Zhengzhou Henan China; ^7^ Center For General Education Westlake University Hangzhou China

**Keywords:** enzyme engineering, human health, machine learning, nanoplastics, PETase, synthetic biology

## Abstract

Microplastics (MPs) accumulation in ecosystem and human organs poses urgent environmental and health risks, yet few enzymes efficiently degrade polyethylene terephthalate (PET) under physiological conditions. We leveraged deep learning to mine unexplored sequence space across 246 million proteins, discovering AhPETase, an evolutionarily distinct hydrolase with low homology (<50% sequence identity) to known PET‐degrading enzymes. This noncanonical biocatalyst efficiently depolymerizes PET at 37°C, outperforming all typical PETases and achieving a 7.76‐fold enhancement over IsPETase, one of the most representative mesophilic PETases. Additionally, engineered variant AhPETase^M1^ retains functional activity for over 20 days under physiological conditions and can degrade post‐consumer PET MPs 34‐fold faster than recombinant human‐derived enzyme MG8 (rMG8) under equal enzyme loading. Critically, it reversed PET‐induced toxicity in human lung and colon cells, establishing the first proof‐of‐concept for enzymatic MPs detoxification.

## Graphical abstract

A deep learning–driven pipeline mining 246 million protein sequences uncovers AhPETase, an evolutionarily distinct PET hydrolase. Engineered variant AhPETase^M1^ degrades post‐consumer PET microplastics under physiological conditions and reverses microplasticinduced cytotoxicity in human lung and colon cells, establishing enzymatic microplastic detoxification as a viable concept.

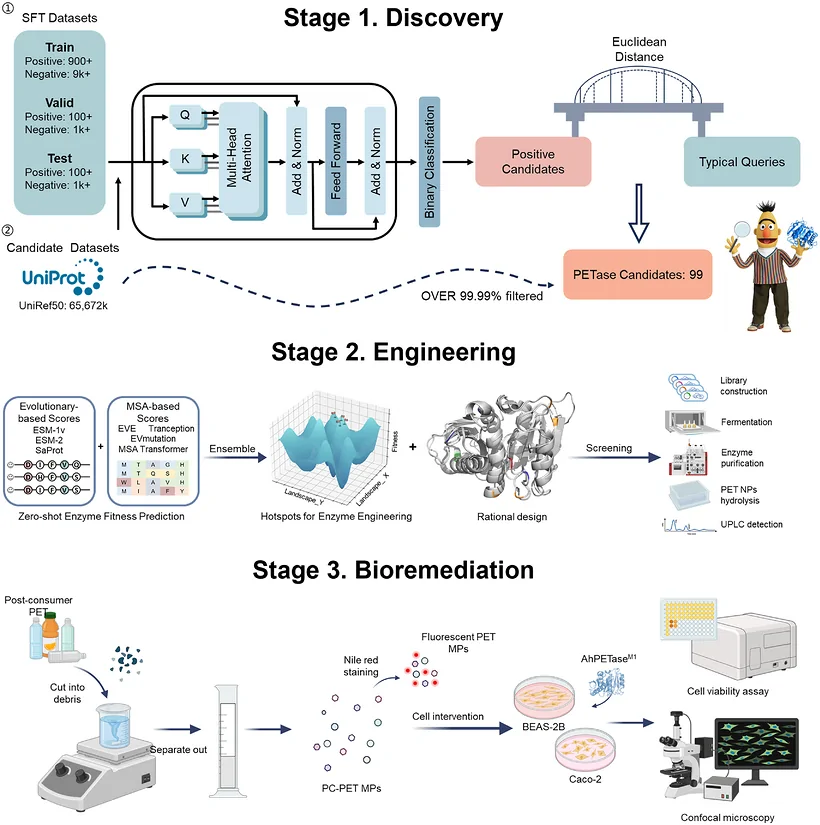

## 1 Introduction

Plastic polymers derived from petroleum are fundamental to modern society due to their versatility and low cost. Current estimates suggest plastic manufacturing will account for approximately 20% of global petroleum use by 2050 [1]. However, the situation of plastic recycling is not optimistic. With less than 10% of consumed plastics being recycled and 79% accumulating in landfills or natural environments [2], an estimated 4.8 to 12.7 million metric tons of plastics enter the world's oceans annually [3]. Over time, plastic waste undergoes fragmentation into micro‐ and nanoplastics—ubiquitous contaminants that pollute ecosystems worldwide [4, 5, 6]. These microplastics (MPs) can enter the human body via the food web, drinking water, and inhalation of airborne particles from household products, raising serious concerns regarding their potential impacts on human health and economic systems [7, 8, 9].

Polyethylene terephthalate (PET), a petroleum‐derived polyester, is one of the most widely produced synthetic plastics globally, with major applications in packaging, textiles, and consumer goods. Its annual production exceeds 82 million metric tons, rendering it a polymer of profound economic importance and environmental concern due to its persistence in ecosystems [10, 11]. While up to 40% of PET is recycled annually through mechanical, chemical, and biochemical processes [12, 13, 14], the remaining 60% accumulates in the environment and poses a threat of irreversible ecological damage [15]. Furthermore, studies have detected PET MPs not only in directly exposed organs (e.g., lungs, intestines) but also in internal organs, including heart and liver [16, 17, 18]. Its predominance among MPs in human stool samples underscores its pervasive exposure [19]. At a cellular level, exposure to PET MPs induces various dose‐dependent cytotoxic effects, such as oxidative stress, inflammatory responses, and DNA damage [20, 21, 22, 23]. Despite these established risks, the development of effective strategies to mitigate or prevent MPs accumulation in living organisms remains an urgent and critically under‐explored challenge.

Multiple PET hydrolases (PETases) have been isolated from the environments [24, 25, 26, 27, 28] and engineered for industrial‐scale PET recycling [29, 30, 31, 32]. While these efforts have successfully enhanced enzymatic activity at elevated temperature, the challenge of mitigating the physiological toxicity of MPs under physiological conditions remains largely unaddressed. This is primarily due to a fundamental limitation: most known PET hydrolases exhibit markedly poor performance against post‐consumer waste under mesophilic and non‐alkaline conditions [32, 33, 34]. Efficient low‐temperature degradation of PET remains a limitation of current enzymatic systems, severely hindering their application in environmental and biomedical contexts. A metagenome‐derived PET hydrolase (MG8) was recently discovered in the human microbiome. While this enzyme demonstrates activity at ambient temperature, its catalytic efficiency remains limited [35]. The development of an efficient PETase capable of low‐temperature degrading is thus essential to elucidate the underlying degradation mechanisms under mild conditions and to inform the rational engineering of more potent mesophilic PETases. Protein language models enable the mining of novel enzymes with enhanced properties by learning evolutionary patterns and sequence‐function relationships from vast datasets [36, 37, 38]. This capability facilitates rapid in silico screening of extensive protein database such as UniProtKB (over 246 million sequences), bypassing complex multi‐omics analysis. This approach holds significant potential for accelerating the discovery of efficient biocatalysts for low‐temperature PET degradation [39, 40].

In this work, we employed the ESM‐1b protein language model to identify AhPETase, a novel PETase from an unconventional evolutionary cluster (<50% identity to known PETases) with superior mesophilic activity and high potential for in vivo MPs removal. By integrating machine learning‐based zero‐shot predictions with structure‐guided rational design, we engineered a superior mutant, AhPETase^M1^. It degraded post‐consumer PET MPs 34‐fold faster than the rMG8, and 1.67‐ and 3.04‐fold faster than the mesophilic PETase, ThermoPETase and CaPETase^M9^, respectively. Molecular dynamics simulations and mutagenesis experiments indicated that enhanced flexibility and reduced steric hindrance in its binding cleft contribute to this improved catalytic activity under physiological conditions. Enzymatic treatment with AhPETase^M1^ significantly reduced the cytotoxicity of post‐consumer PET MPs in human cell lines. Cell viability was markedly increased in BEAS‐2B cells (from 74.63 ± 4.02% to 110.57 ± 2.18%) and Caco‐2 cells (from 69.78% ± 2.98% to 95.18% ± 0.70%). Multi‐omics analysis elucidated the potential underlying molecular mechanisms, revealing associated alterations in genes and proteins involved in cellular homeostasis and fate decisions. In summary, this study identified and engineered a highly active mesophilic PETase, and provided a perspective for mitigating the adverse effects of microplastic accumulation in living organisms.

## 2 Results

### 2.1 Identifying AhPETase With Enhanced Catalytic Activity Under Physiological Conditions Using an ESM‐Derived Strategy

To identify superior PETase candidates for engineering efficient PET degradation under physiological conditions, we developed a pipeline combining in silico prediction with wet‐lab validation. First, we fine‐tuned ESM‐1b to learn semantic representations of PET hydrolase and perform accurate classification, using over 900 PETase sequences (positive dataset) from prior literature and UniProt, along with randomly sampled non‐PETase sequences (negative dataset) from SWISS‐PROT. The model's robustness and fine‐tuning efficiency were confirmed by five‐fold cross‐validation (Table S1) and t‐Distributed stochastic neighbor embedding (t‐SNE) analysis (Figure S1). We then generated high‐dimensional embeddings of the Uniref 50 dataset (numerical representations of sequence features) by processing through the fine‐tuned model. To identify candidate PETases, we employed a multi‐query approach based on structural and evolutionary features of five query enzymes (QEs) (BhrPETase [28], Cut190 [41], IsPETase [27], LCC [24], TfCut2 [42]). Candidates were ranked by mean Euclidean distance and hit frequency relative to these reference enzymes. In silico screening eliminated more than 99.99% of the sequences (Figure 1a), yielding ten high‐confidence and soluble candidates for further validation (Figures S2–S4 and Table S2).

**FIGURE 1:**
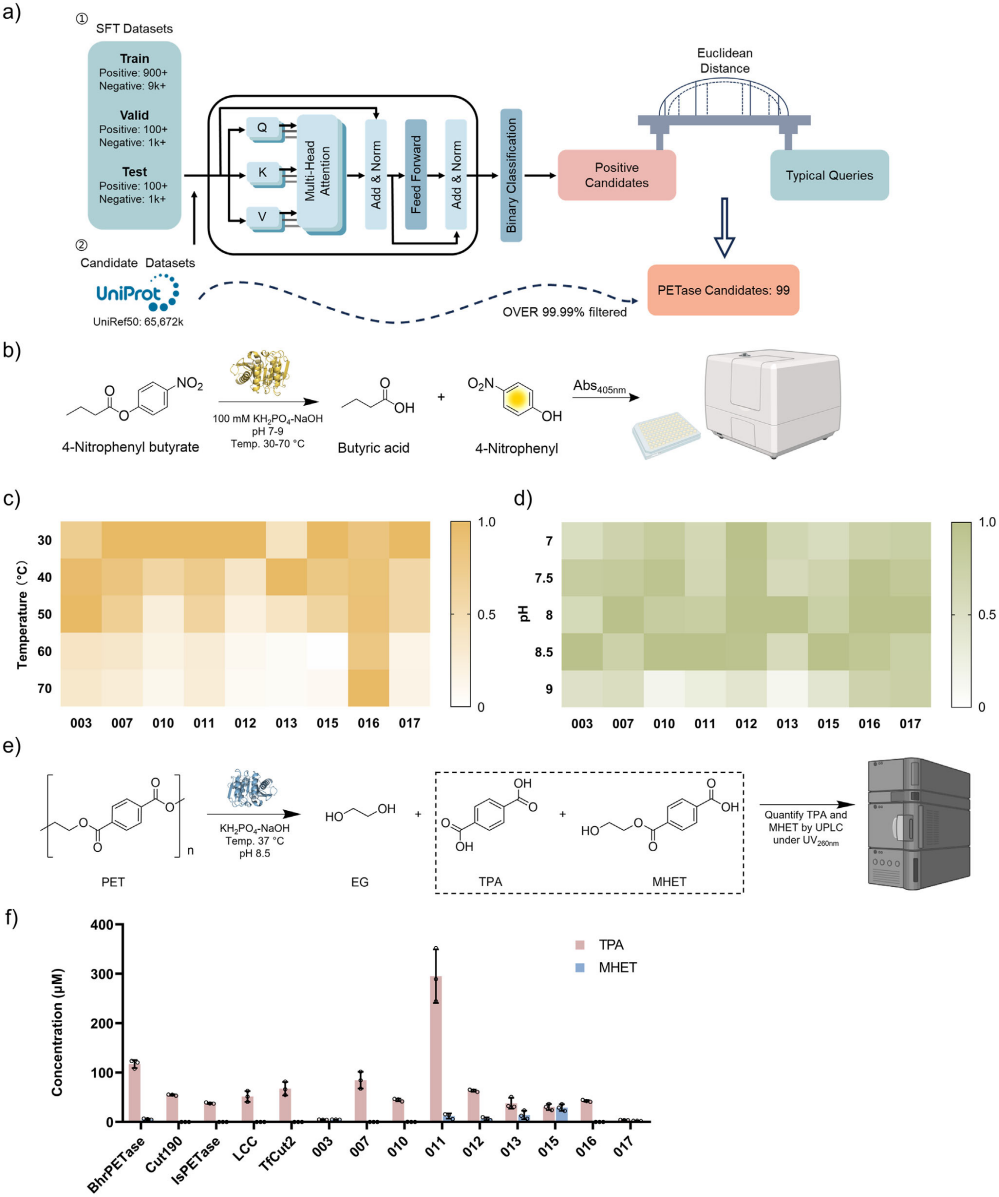
Discovery and characterization of PETase candidates. a) ESM‐1b was fine‐tuned for binary classification to identify enzyme candidates via latent space projection. Target localization is achieved by computing Euclidean distances between multi‐query and positive candidate embeddings. b) High‐throughput functional screening platform using 4‐nitrophenyl butyrate (*p*NPB) for rapid activity detection. c) Temperature‐activity profile of the novel PETase from 30 to 70°C. d) pH‐activity profile of the expressed PETase between pH 7 and 9. e) Workflow for assessing PET degradation by the novel PETase, with UPLC detection of terephthalic acid (TPA) and mono(2‐hydroxyethyl) terephthalate (MHET) as main products. f) Comparative activity of query enzymes (QEs) and candidates on semi‐crystalline PET (CryPET) at 37°C. Data are presented as mean ± s.d. (n = 3).

We next assessed catalytic activity of selected candidates against 4‐nitrophenyl butyrate (*p*NPB) across a temperature range (30 – 70°C) and various pH values in phosphate buffer (Figure 1b). Optimum activity for 60% of the candidates was observed at 30°C and pH 8.0‐8.5 (Figure 1c, d). The depolymerization performance of selected candidates was benchmarked against QEs using semi‐crystalline PET (CryPET) powder as substrate at pH 8.5 and 37°C (Figure 1e) [30, 32, 43]. As shown in Figure 1f, nine out of ten candidates showed catalytic activity at physiological temperature (37°C). Notably, AhPETase (Candidate 011: A0A239PER3), identified as a dienelactone hydrolase from *Asanoa hainanensis*, demonstrated the highest catalytic activity. Its depolymerization efficiency surpassed all QEs, including a 7.76‐fold improvement over IsPETase, one of the most representative mesophilic PETases. These results position AhPETase as a promising candidate for further engineering and analysis.

### 2.2 Defining AhPETase as a Unique PET Hydrolase Different From Known PETases

Distinct from all known QEs, AhPETase displays unique evolutionary and sequence divergence. Our phylogenetic analysis, which incorporated nine new functional candidates and 14 known type I and II PETases, revealed that AhPETase along with candidates 007, 016, and 017 form a novel clade, separate from established mesophilic (e.g., IsPETase) and thermophilic (e.g., LCC) lineages (Figure 2b). Although AhPETase shares conserved secondary structures with other PETases (Figures S5 and S6) [44, 45, 46], the amino acids lining its substrate‐binding cleft differ significantly. Among known PETase‐like enzymes, the residues of subsite I and II determine the binding mode of the first MHET moiety and serve as a classification basis (Figure 2a). Unlike the characterized PETases, which conserve a key Y/F‐Q‐M‐W motif in subsite I, AhPETase possesses a distinct L61‐S93‐M130‐H154 composition, suggesting a unique MHET binding mode for its distinct combination.

**FIGURE 2:**
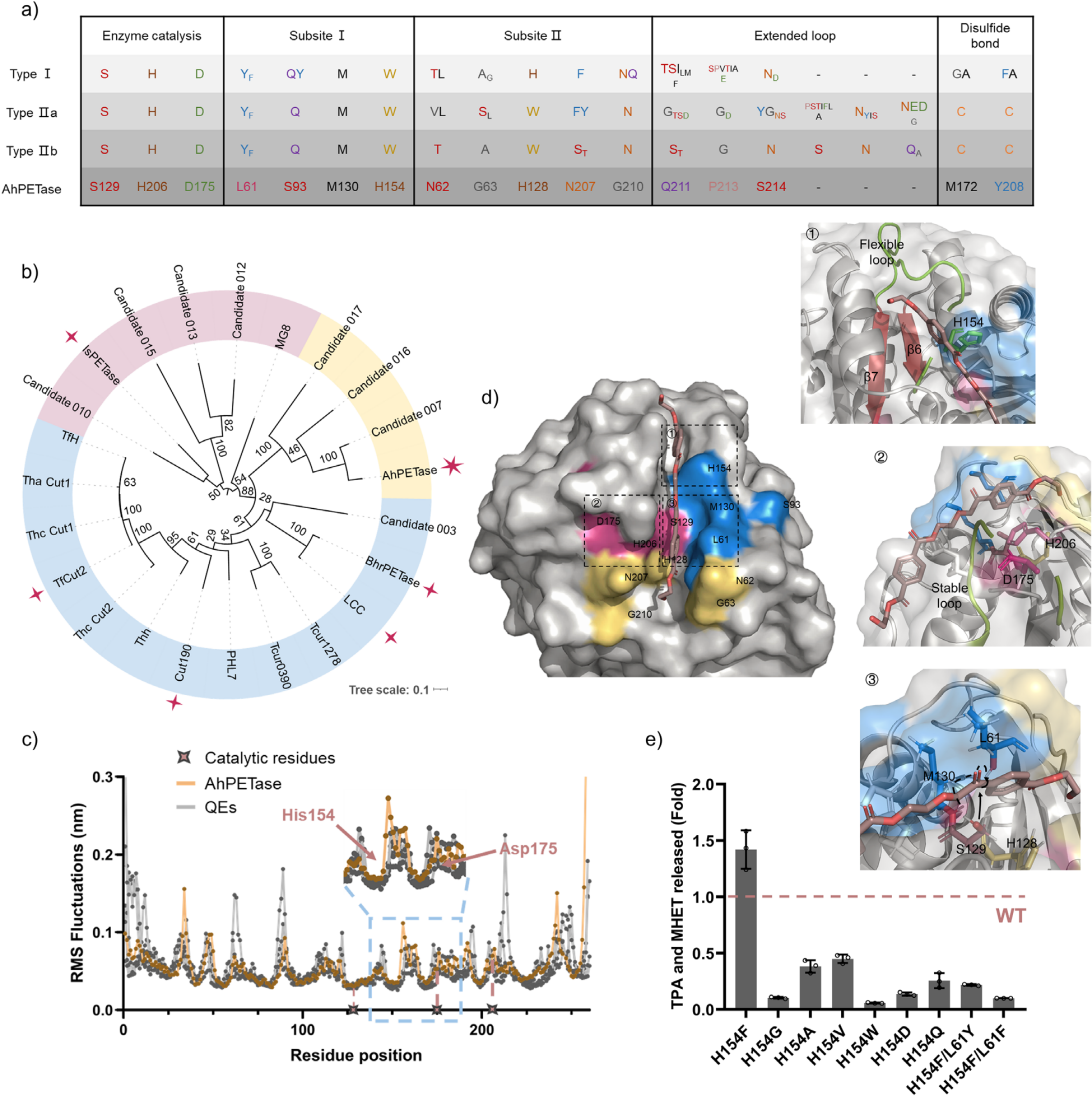
Identification and structural analysis of AhPETase. a) Sequence alignment of key residues in PET hydrolases. Residue variations in subsite I, subsite II, the extended loop, and the disulfide bond are color‐coded for AhPETase, type I, type IIa, and type IIb enzymes. b) Phylogenetic analysis of candidate PETases and known PET hydrolases. Queries and AhPETase are marked by a magenta four‐point star and hexagram, respectively. Type I (blue), type II (red), and the noncanonical candidate cluster (yellow) are highlighted. Bootstrap values (out of 100) are shown. c) Structural dynamics from molecular dynamics simulations. Root‐mean‐square fluctuation (RMSF; nm) of Cα atoms for AhPETase and five query enzymes (QEs). Key PET‐binding loops and the catalytic loop are indicated (blue circle). d) Predicted binary complex of AhPETase with 2‐HE(MHET)_2_, generated by Boltz‐2, depicting substrate binding in the catalytic pocket. The catalytic triad (magenta), subsite I (blue), and subsite II (yellow) residues are shown; key residues are displayed as sticks in the detailed view. e) PET hydrolytic activity of enzyme variants. Total amounts of released products (TPA and MHET) are shown. Pink dash line represents the performance of wild type. Data are presented as mean ± SD (n = 3).

To elucidate the structural basis for AhPETase's superior activity under mesophilic conditions, we analyzed the flexibility of its substrate‐binding and catalytic loops. This analysis was guided by the established principle that optimal PETase activity arises from a balance of loop flexibilities: the PET‐binding loop provides flexibility for efficient capture, coupled with a stable catalytic loop for efficient hydrolysis [47]. This principle is exemplified by the thermally stable variant HotPETase, which was engineered from IsPETase via 21 mutations designed to stabilize the catalytic loop and enhance the flexibility of the substrate‐binding loop [32]. Root mean square fluctuation (RMSF) analysis highlighted a distinct flexibility profile in AhPETase compared to QEs. Specifically, the stability of Asp175‐containing catalytic loop was comparable to QEs, while the His154‐containing substrate‐binding loop was more flexible (Figure 2c). We propose that this specific rigidity‐flexibility balance facilitates optimal substrate positioning by the flexible loop and ensures catalytic efficiency through a stable triad, explaining the enzyme's superior performance at low temperature.

AhPETase features a long and broad substrate‐binding cleft (Figure 2d). Within subsite I, the aromatic residue His154, occupying the position of a conserved tryptophan in other PETases, forms a typical T‐stacked configuration with the benzene ring of the substrate, suggesting an analogous mechanism for substrate stabilization. Mutating His154 to non‐aromatic residues severely reduced activity (Figure 2e), confirming the critical role of this aromatic side chain in positioning the first MHET moiety. Notably, introducing an H154W mutation reduced enzymatic activity by 17.27‐fold. In contrast, the H154F mutant displayed a 1.42‐fold increase in TPA and MHET formation. We speculate that the large steric bulk of the conserved tryptophan introduces unfavorable steric hindrance, impeding optimal substrate binding. Conversely, the smaller side chains of histidine and phenylalanine likely reduce steric conflict and permit greater conformational flexibility, which accounts for their enhanced activity under mesophilic condition. This interpretation is further supported by protein engineering studies in which mutation of the conserved tryptophan to histidine enhanced degradation efficiency in other PETases [33, 48].

Within subsite I, Leu61 and Met130 are positioned to assist in forming the oxyanion hole, which polarizes the ester bond and stabilizes the tetrahedral intermediate during nucleophilic attack by the catalytic triad [49]. In contrast to most type I/II PETases, where a Tyr or Phe residue typically occupies this position to anchor the terephthalic ring in a strained conformation [50, 51], the Leu61 residue in AhPETase significantly reduces steric hindrance. Furthermore, the main chain atoms of Leu61 appear to form hydrogen bonds with the substrate (Figure S7). Mutation of Leu61 to amino acids with bulkier side chain (Tyr and Phe) reduced its activity tremendously (Figure 2e), which indicates that the reduced steric hindrance within the substrate‐binding loop is likely responsible for a beneficial effect on AhPETase activity under mesophilic condition.

### 2.3 Engineering AhPETase With Improved Catalytic Efficiency for PET MPs at 37°C

Given the distinct sequence space of AhPETase compared to other reported ones, we employed both machine learning‐assisted zero‐shot prediction and structure‐based rational design to engineer variants with improved catalytic activity under mesophilic conditions (Figure 3a). Based on site‐saturation mutational effect predictions and fitness voting, His154, Asp163, Tyr170 and Met172 were identified as hotspots. Concurrently, structure‐based rational design was employed to introduce stabilizing noncovalent bonds (e.g., L90T, P156T) and a disulfide bond (T202C/S248C). We also mutated specific residues in AhPETase's subsites I and II to their conserved counterparts in other PETases (e.g., L61F, S93Q, G210N, etc.) to probe the impact of these substitutions. Screening of the mutagenesis library was performed by incubating purified enzyme variants with uniform CryPET MPs and quantifying the released TPA and MHET via UPLC. Subsequently, beneficial mutations were combinatorially assembled to generate synergistic variants, whose performance is detailed in Figure 3b.

**FIGURE 3:**
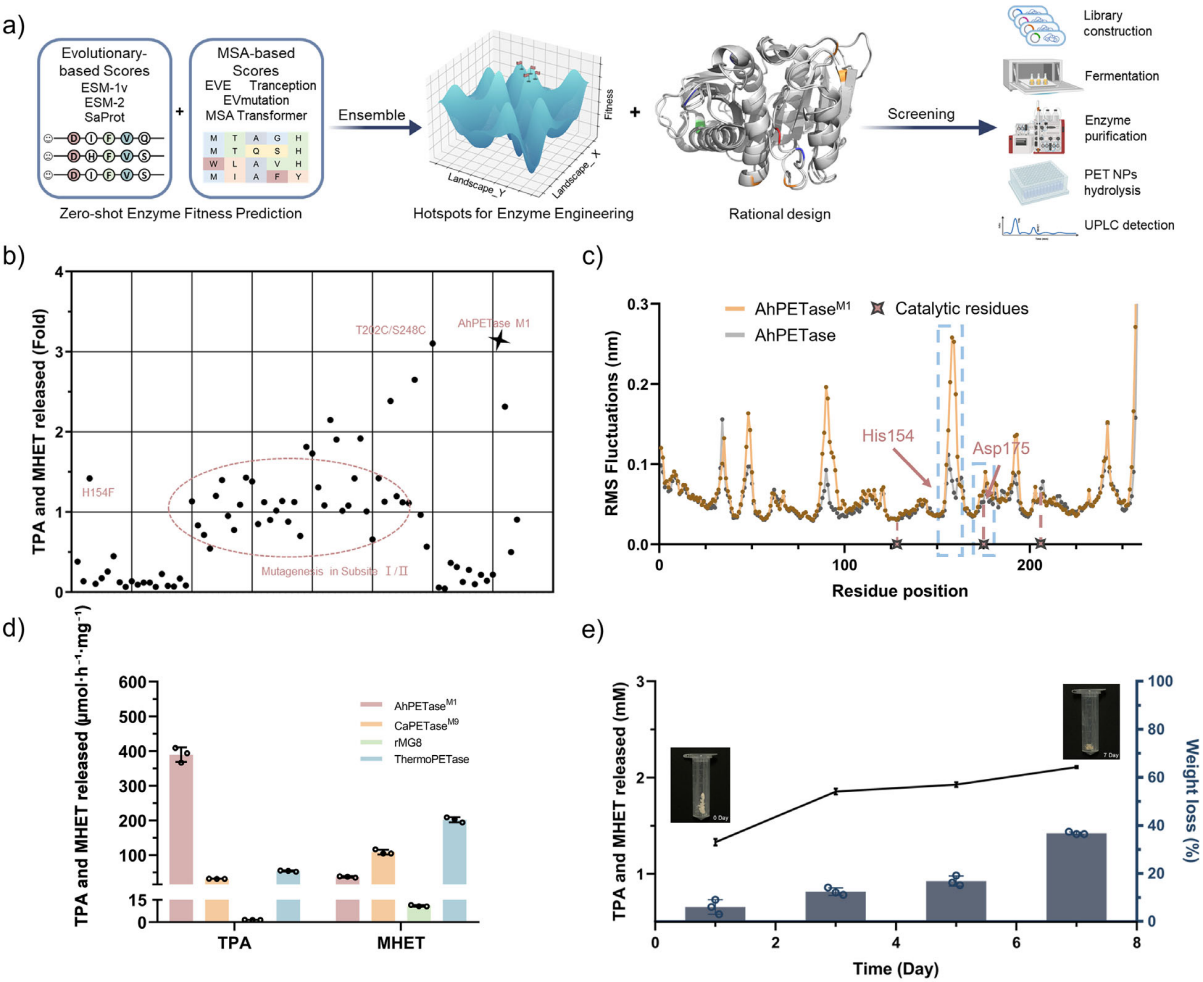
Engineering of AhPETase for Pc‐PET degradation. a) Engineering strategy combining rational design with computational predictors of mutational effects, including evolutionary (ESM‐1v, ESM‐2, SaProt) and MSA‐based (EVE, Tranception, EVmutation, MSA Transformer) models. b) Screening of single and combinatorial AhPETase mutants. Key variants are highlighted (pink). Data points represent the average fold‐change in total product (TPA and MHET) release, normalized relative to the wild‐type baseline within each experimental batch, from triplicate reactions. c) Structural flexibility comparison. Molecular dynamics simulations show Cα root‐mean‐square fluctuation (RMSF) for wild‐type AhPETase and the engineered variant AhPETase^M1^. d) Degradation of CryPET MPs at 37°C by AhPETase^M1^ vs. other mesophilic PETase mutants at same enzyme loading. Data represent mean ± SD. (n = 3). e) Time‐course degradation over 7 days at 37°C. Top: Representative images showing the physical changes of Pc‐PET MPs from day 0 to day 7. Bottom: Quantification of soluble products (TPA, MHET) by UPLC (line, mean ± SD, n = 3) and degradation rate by weight loss (bars).

The highest cumulative production of TPA and MHET was observed for variant AhPETase^M1^ (H154Y), which achieved a 3.15 ± 0.09‐fold increase over wild‐type activity. Maximal TPA production rate was shown in the variant AhPETase^T202C/S248C^ with a 4.27‐fold increase (Figure S8a), whereas the double mutant AhPETase^H154F/G210S^ showed a 28.75‐fold enhancement in MHET production (Figure S8b). Notably, the introduction of the T202C/S248C disulfide bond into other beneficial mutants severely impaired the total product yield. MD simulation revealed that the H154Y mutation significantly enhanced the flexibility of the PET‐binding loop, which contributes to its improved catalytic efficiency under mesophilic conditions. Consequently, AhPETase^M1^ was selected as the optimal candidate due to its superior degradation rate of CryPET MPs at 37°C (Figure 3d). It exhibited a total product (TPA and MHET) liberation rate of 427.06 µmol·h^−^^1^·mg^−^^1^, representing a 34.06‐fold enhancement over rMG8. It also outperformed other engineered IsPETase variants, with activities 1.67 and 3.04 times greater than those of ThermoPETase and CaPETase^M9^, respectively. Notably, AhPETase^M1^ retained its catalytic activity for over 20 days (Figure S9). This prolonged stability provides a strong foundation for further investigating its potential detoxification effects under physiological conditions.

To evaluate the performance of AhPETase^M1^ on consumer PET products, we prepared MPs from post‐consumer PET (Pc‐PET MPs, in Figure S10) derived from beverage bottles and fruit baskets. We performed a 7‐day degradation assay with Pc‐PET MPs at 37°C (Figure 3e). The reaction achieved 63.03% cumulative products yield within the initial 24 h and released 2.11 ± 0.03 mM TPA and MHET in total. Progressive depolymerization led to significant volumetric shrinkage, visible yellowing, and aging of the Pc‐PET MPs, ultimately achieving 36.70% mass loss‐based degradation.

### 2.4 Evaluating Physiological Toxicity of Pc‐PET MPs and Potential Detoxification Effect of AhPETaseM1

We next evaluated the detoxification potential of AhPETase^M1^ against the toxicity induced by Pc‐PET MPs (Figure 4a). To validate the feasibility of physiological detoxification, we assessed the catalytic performance of AhPETase^M1^ in MEM basal cell culture media (∼pH 7.4, 37°C). AhPETase^M1^ exhibited robust and sustained catalytic activity, with the final degradation products (TPA and MHET) reaching approximately 7 mM by day 7 (see Figure S11 for more details). To establish a toxicity baseline, we characterized the adverse effects of Pc‐PET MPs on BEAS‐2B and Caco‐2 cell lines, selected as the model systems representing the primary exposure routes and major sites of Pc‐PET MPs accumulation [18]. Imaging of Nile red‐stained fluorescent Pc‐PET MPs revealed distinct, cell‐type‐specific internalization patterns (Figure 4b, c). BEAS‐2B cells internalized MPs into perinuclear aggregates, while Caco‐2 cells displayed a diffuse cytoplasmic distribution. The proliferation morphology of two cell lines and aggregation behavior of Caco‐2 cells remained unaffected by MPs exposure.

**FIGURE 4:**
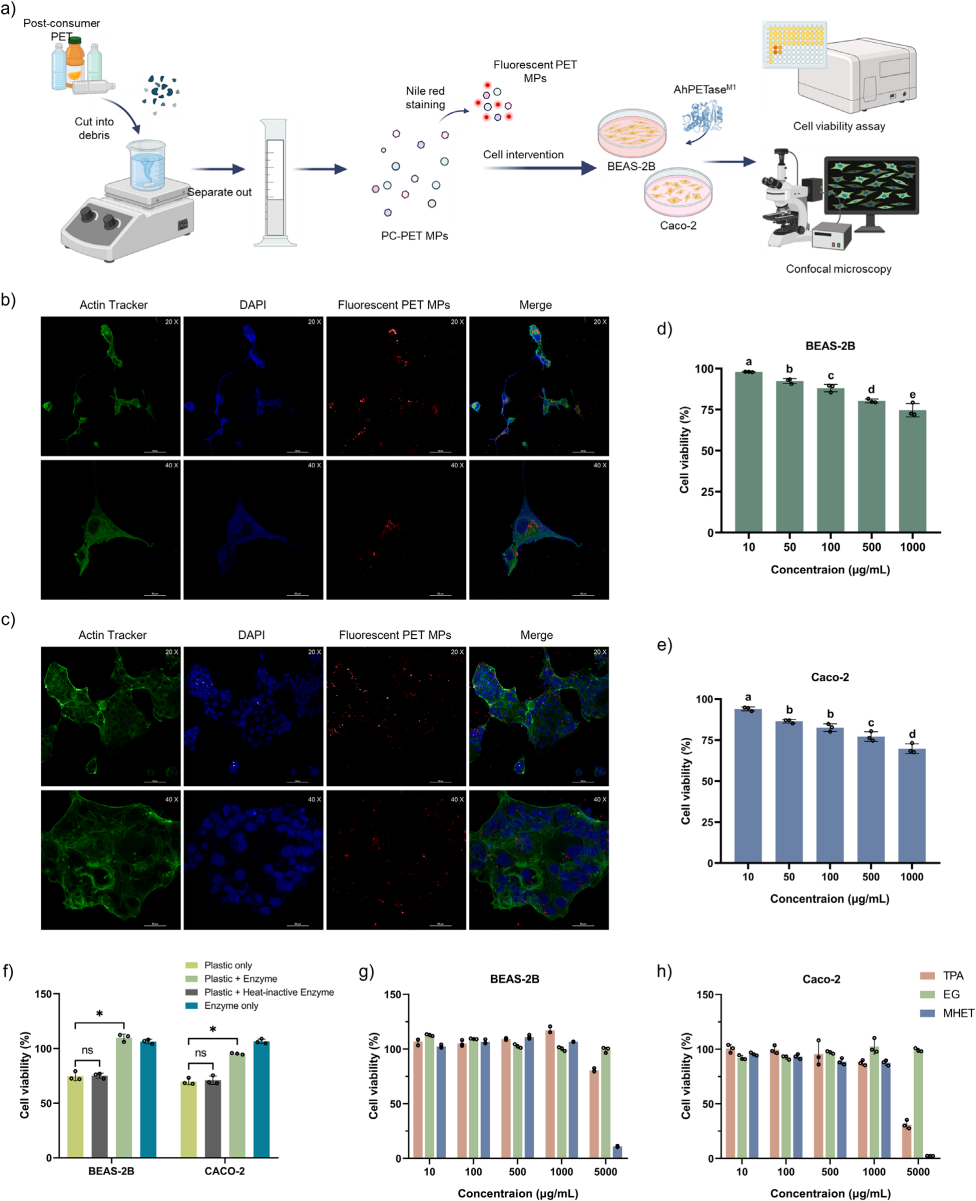
In vitro toxicological assessment of Pc‐PET MPs. a) Schematic workflow for the preparation of Pc‐PET MPs and subsequent cytotoxicity evaluation. b) Cellular uptake of fluorescent Pc‐PET MPs. Confocal microscopy images show MPs localization (red) in BEAS‐2B cells. Nuclei are stained with DAPI (blue) and actin filaments with phalloidin (green). c) Cellular uptake of fluorescent Pc‐PET MPs. Confocal microscopy images show MPs localization (red) in Caco‐2 cells. Nuclei are stained with DAPI (blue) and actin filaments with phalloidin (green). d) Cytotoxicity of Pc‐PET MPs on BEAS‐2B cells across a range of concentrations. Data are presented by mean ± s.d. (n = 3; *p < 0.05, one‐way Welch's ANOVA with Dunnett's T3 test) e) Cytotoxicity of Pc‐PET MPs on Caco‐2 cells across a range of concentrations. f) Attenuation of Pc‐PET MPs‐induced cytotoxicity by co‐treatment with AhPETase^M1^ in both BEAS‐2B and Caco‐2 cell lines. g) Cytotoxicity of major Pc‐PET MPs degradation products, EG, TPA and MHET, on BEAS‐2B cells at varying concentrations. h) Cytotoxicity of major Pc‐PET MPs degradation products, EG, TPA and MHET, on Caco‐2 cells at varying concentrations. Data are presented by mean ± s.d. (n = 3; *p < 0.05, one‐way ANOVA with Tukey's test in 4e and 4f).

Pc‐PET MPs induced dose‐dependent anti‐proliferative effects on BEAS‐2B and Caco‐2 cells following 48 h exposure (Figure 4d, e; Figure S12). Caco‐2 cells demonstrated higher sensitivity, exhibiting reduced viability (94.06% ± 1.19%) at 10 µg/mL. In contrast, a comparable reduction in BEAS‐2B viability (92.38 ± 1.50%) required a higher concentration of 50 µg/mL. At the highest concentration tested (1,000 µg/mL), viability was significantly reduced to 74.63 ± 4.02% in BEAS‐2B cells and 69.78 ± 2.98% in Caco‐2 cells. Notably, co‐treatment with 1,000 µg/mL Pc‐PET MPs and 10 µM AhPETase^M1^ restored cell viability to 110.57% ± 2.18% in BEAS‐2B cells and 95.18 %± 0.70% in Caco‐2 cells. In contrast, heat‐inactivated AhPETase^M1^ failed to reverse the adverse effects of the MPs on cell viability. These results clearly demonstrate the potential detoxifying effect of AhPETase^M1^ (Figure 4f).

To assess the cytotoxicity of the principal PET degradation products, BEAS‐2B and Caco‐2 cells were treated individually with ethylene glycol (EG), terephthalic acid (TPA), or mono(2‐hydroxyethyl) terephthalate (MHET) across a concentration range of 10 to 5,000 µg/mL. EG exhibited negligible cytotoxicity in both cell lines, with viability remaining largely unaffected even at 5,000 µg/mL (Figure 4g, h). In contrast, TPA induced concentration‐dependent cytotoxicity, significantly reducing Caco‐2 cell viability to 87.49% ± 2.37% at 1000 µg/mL and to 31.00 ± 3.91% at 5000 µg/mL. BEAS‐2B cells exhibited greater resistance to TPA, maintaining 80.56 ± 1.72% viability at the highest concentration (5,000 µg/mL). Additionally, both cell lines exhibited high tolerance to MHET at concentrations up to 1,000 µg/mL. Given that enzymatic treatment of Pc‐PET MPs generates EG, TPA, and MHET at concentrations within these established safety thresholds, the observed restoration of cell viability following co‐treatment with AhPETase^M1^ is likely attributable to the reduction in cellular exposure to the Pc‐PET MPs (Table S7).

## 3 Discussion

While plastic recycling offers significant economic benefits, greater attention must be directed toward the environmental and ecological damage caused by unrecycled plastic waste, which persists in natural environments and living organisms posing serious ecological and health risks. Predators are exposed to MPs through the food chain, while vegetables directly absorb these particles from the air through their leaves [52]. As a persistent environmental pollutant, MPs have been detected even within the male reproductive system and will impact human health for generations [53]. Conventional engineering efforts have primarily focused on thermophilic PETases for industrial applications (e.g., LCC‐A2 [54], TurboPETase [55]), leaving the challenge of low‐temperature PET degradation largely unaddressed due to the absence of highly active mesophilic PETases. Our discovery and engineering of AhPETase^M1^, which demonstrates exceptional performance at physiological temperature, provides a foundational solution for the bioremediation of environmentally persistent PET waste.

As previously demonstrated, the capacity for PET degradation is primarily governed by the flexibility of substrate‐binding loops and the stability of the catalytic loop [56]. Enhanced flexibility in PET‐binding loop containing His154 generally correlates with improved substrate capture efficiency. This structural feature likely explains the superior performance of AhPETase compared to all QEs at lower temperature. We further proved this conclusion by mutating His154 to Tyr, which improved the flexibility of this loop, and significantly enhanced AhPETase's activity by 3.15‐fold. In addition to loop dynamics, steric hindrance within the PET‐binding loop is a critical determinant of low‐temperature PET degradation activity. Replacing the unique PET‐binding loop residue Leu61 with the bulkier Tyr or Phe found in type I/II PETases significantly impaired enzymatic activity, demonstrating the detrimental effect of increased steric hindrance on mesophilic activity. These results highlight a previously underexplored aspect of loop steric effects, offering new considerations for future PETase engineering aimed at enhancing low‐temperature catalytic efficiency.​

In this study, we compared the PET MPs degradation efficiency of a human saliva metagenome‐derived PET hydrolase (MG8) with the engineered AhPETase^M1^. As the first PETase identified from the human metagenome [35], the presence of MG8 may represent an evolutionary response to the accumulation of synthetic polymers such as PET within the human body, ​​suggesting that enzymatic strategy may serve as a feasible mechanism for mitigating internal health risks. AhPETase^M1^ demonstrated a 34‐fold enhancement in activity over rMG8. Co‐treatment with AhPETase^M1^ significantly enhanced cell viability in both lung (BEAS‐2B) and intestinal (Caco‐2) cell lines compared to MPs exposure alone. To elucidate the cell‐specific mechanisms underlying this detoxification, we performed a multi‐omics analysis (transcriptomics and proteomics; Figure S13). In BEAS‐2B cells, the co‐treatment with AhPETase^M1^ and MPs, compared to MPs alone, induced marked shifts in the transcriptomic profiles of genes related to endocytosis, oxidative stress, and lipid metabolism. In contrast, Caco‐2 cells exhibited alterations in transcripts involved in immune regulation and the cell cycle. These transcriptomic findings were corroborated by corresponding proteomic changes, notably in cytoskeletal components in BEAS‐2B cells and ribosomal proteins in Caco‐2 cells. Collectively, the results indicate that AhPETase^M1^ treatment mitigates MPs‐induced toxicity by modulating key transcriptional and translational programs governing cellular homeostasis and fate decisions, thereby contributing to the observed recovery of cell viability. Together, further investigation is needed to elucidate the detailed mechanisms underlying this enzymatic remediation process.

The recent development of PETases with activity at lower temperatures highlights their potential for degrading microplastics under physiological conditions [29, 57]. As a proof‐of‐concept, our study demonstrates that enzymatic treatment can mitigate MP‐induced toxicity. However, translating this exogenous biocatalyst into complex in vivo mammalian systems presents profound pharmacological and practical challenges. First, introducing recombinant bacterial proteins into mammalian hosts risks off‐target distribution, immune recognition, and rapid proteolytic degradation [58, 59]. In addition, integrating these enzymes into sophisticated, tissue‐specific targeted delivery vehicles remains a formidable pharmacological hurdle [60]. Furthermore, while the health risks of microplastics are recognized global concerns, the precise quantification of their accumulation in human tissues and the establishment of definitive clinical biomarkers for chronic in vivo toxicity remain ongoing challenges in epidemiological research [61, 62, 63]. The current lack of standardized, quantifiable indicators for in vivo MPs toxicity makes it premature to design rigorous translational animal studies or to establish standardized dosing regimens for complex delivery systems. Despite these translational hurdles, this study establishes the biochemical feasibility of enzymatic microplastic degradation under physiological conditions, providing a biocatalytic toolkit ready for in vivo application once definitive clinical metrics are established.

In conclusion, by mining 246 million sequences via an AI‐driven pipeline, we discovered AhPETase, a noncanonical biocatalyst that outperforms benchmark PETases at 37°C. Guided by the unique flexibility and reduced steric hindrance of its PET‐binding loops, we engineered AhPETase^M1^, an optimized variant that maintained catalytic activity for over 20 days and efficiently degraded post‐consumer PET microplastics under physiological conditions. Furthermore, this enzymatic treatment successfully reversed microplastic‐induced cytotoxicity in human BEAS‐2B and Caco‐2 cell lines. Ultimately, this work elucidates the molecular basis of low‐temperature PET depolymerization and provides the first proof‐of‐concept for the physiological detoxification of microplastics.

## 4 Experimental Section

### 4.1 Dataset Collection, Model Fine‐Tuning and Candidates Screening

The positive dataset was directly collected by searching for ‘Poly (ethylene terephthalate) hydrolase’ within UniProtKB dataset (Until March, 2024), and we finally got 927 valid sequences (14 reviewed in Swiss‐Prot and 913 unreviewed in TrEMBL). The negative dataset was prepared by randomly sampling from the whole Swiss‐Prot (screened by label ‘NOT Poly (ethylene terephthalate) hydrolase’).

In the model fine‐tuning process, we performed five‐fold cross‐validation to assess the model's robustness. The positive dataset was divided into five folds, with the negative dataset remaining constant for both training and testing. In each iteration, one positive fold served as the test set, while the remaining folds were used for training. We calculated accuracy, precision, recall, F1 – score, and ROC—AUC based on this setup.

With the fine‐tuned binary classification model, we used Uniref 50 database as candidate pool to discovery potential PET hydrolases as positive sequences. We collected five typical and efficient polyethylene terephthalate hydrolases (BhrPETase, Cut190, IsPETase, LCC, TfCut2) as described in Table S3 and used them as query sequences for cooperative search. Subsequently, Euclidean distance between positive candidates and five typical queries were calculated by representations from fine‐tuned model within the high‐dimensional semantic space. For each query, the top 20 sequences with the closest Euclidean distance were identified. The quality of these different candidate sequences was then assessed based on two factors: the frequency with which they were located by the various queries and the mean Euclidean distance between them and the queries. More specific details in approaches, configuration, and iterations were described in Notes S4 and S5.

### 4.2 Phylogenetic Analysis, Sequence Identity and TM‐Score

Phylogenetic analysis was performed by MEGA 12 [64], sequences used were described above. The phylogeny was inferred using the Maximum Likelihood method and the Jones‐Taylor‐Thornton (1992) model of amino acid substitutions. The tree with the highest log likelihood (−8,714.97) is presented. The percentage of replicate trees in which the associated taxa clustered together is shown at the nodes, with the number of bootstrap replicates (107) determined adaptively. Initial tree(s) for the heuristic search were obtained by applying the Neighbor‐Joining method to a matrix of pairwise distances estimated using Jones‐Taylor‐Thornton (1992) model [64, 65, 66]. The analytical procedure encompassed 23 amino acid sequences with 432 positions in the final dataset. Evolutionary analyses were conducted in MEGA12 utilizing up to 4 parallel computing threads.

Sequence identity and TM‐Score were used to evaluate the relationship within sequence and structures between queries and candidates. The sequence identity between each query and candidates were calculated by using Align tool provided by UniProt website (Advance parameters: order = ‘same as input’, iterations = 5). And the TM‐Score between queries and candidates were calculated by TM‐align, a structure alignment program written by Yang Zhang lab [67]. Since none of the newly identified candidates possessed available structural information, we predicted their structures with AlphaFold 2.2 [68].

### 4.3 An Ensemble ML Model for Zero‐shot Protein Fitness Prediction

To engineer the brand‐new PETase, we adopted an ensemble strategy integrating protein language models (PLMs) and sequence density models for zero‐shot fitness predictions. Specifically, ESM‐1v, ESM‐2, and Saprot served as PLMs‐based fitness predictors (Note S6). EVmutation functioned as an evolutionary coupling model, and EVE acted as a latent generative sequence model. Tranception and MSA Transformer were utilized as hybrid PLMs, enhancing local evolutionary patterns within global evolutionary contexts. The individual scores from the above models underwent z‐score normalization to achieve a comparable scale with zero mean and unit variance. Subsequently, these normalized scores were aggregated through summation to yield the final variant effect scores:

$$f_{\left(x\right)}=\sum \left[{\bar{S}}_{PLMs}\left(x\right)+{\bar{S}}_{EVmutation}\left(x\right)+{\bar{S}}_{EVE}\left(x\right)+{\bar{S}}_{HybridPLMs}\left(x\right)\right]$$

where, $\bar{s}$ represents for all normalized scores of corresponding models.

Different mutants of the target protein were ranked according to these final scores. Hotspots for further enzyme engineering were identified by analyzing the frequency of mutated positions among the top 20 ranked variants.

### 4.4 Molecular Docking

The structure of AhPETase was predicted by AlphaFold 2.2. The validation of 3D modelled structures over stereo chemical quality with residue‐by‐residue geometry by using Procheck [69]. Subsequently, the substrates (2‐HE(MHET)2) were docked into the AhPETase by Boltz‐2 [70]. Based on the crystal structure of the lsPETase‐(1‐(2‐hydroxyethyl) 4‐methyl terephthalate) complex [49], the optimal docking conformation was selected for subsequent analysis by comprehensively evaluating binding pattern.

### 4.5 MD Simulations

The 3D structures of BhrPETase (PDB: 7EOA), Cut190 (4WFI), IsPETase (5XFY), LCC (4EB0), and TfCut2 (4CG1) were obtained from the Protein Data Bank, while AhPETase^M1^’s structure was predicted using AlphaFold 2.2. All protein models underwent protonation state preparation at pH 8.5 via PROPKA3 calculations. Each protein was positioned centrally within a cubic box maintaining 1 nm clearance from edges, subsequently solvated with water molecules, and neutralized with 0.1 M NaCl. Molecular dynamics simulations were executed using GROMACS 2022.2 [71] with the amber99sb‐ildn force field [72] under periodic boundary conditions. Systems maintained constant temperature (310 K, Berendsen thermostat) and pressure (1.0 bar, Parrinello‐Rahman barostat), employing 1.2 nm cutoffs for both van der Waals and electrostatic interactions, while bond lengths were constrained using the LINCS algorithm [73].

Following energy minimization, systems underwent 0.1 ns equilibration prior to sequential 1 ns NVT and 1 ns NPT ensemble simulations. Long MD runs were conducted at varying durations: 100 ns for AhPETase, TfCut2 and Cut190; 150 ns for BhrPETase; and 200 ns for AhPETase^M1^, IsPETase and LCC to achieve systems’ stability. The conformational stability of the protein was assessed by calculating backbone root‐mean‐square deviation (RMSD) relative to the energy‐minimized starting structure, with least‐squares superposition performed on Cα atoms during the MD simulations. Local structural flexibility was quantified through per‐residue root‐mean‐square fluctuation (RMSF) analysis using the last 10 ns of equilibrated trajectories, following backbone (N, Cα, C) alignment to the reference structure with periodic boundary condition correction.

### 4.6 Cloning and Library Construction

The genes encoding five typical wildtype PETases, new candidates and mutants (ThermoPETase, CaPETase^M9^, MG8) were commercially synthesized and cloned into corresponding plasmids (Table S4) by GenScript Biotech Corporation. Then the recombinant plasmids were transformed into *E. coli* C41 (DE3) by heat‐shock method and stored for further utilization. The mutant library was constructed by primers described in table S5, single colonies from a fresh transformation were used to inoculate and kept in 96‐deep‐well plate for further fermentation. According to Eiamthong et al. [35], MG8 from human saliva metagenome formed insoluble inclusion bodies in *E. coli* BL21 (DE3), implementation of the SUMO‐thioredoxin dual‐tag system enabled successful soluble expression and showed no signs of significant impairment of activity. (Figure S14).

### 4.7 Protein Expression and Purification

Single colony of freshly activated cells containing target gene were cultured in 6 mL LB media supplemented with corresponding antibiotics (25 µg/mL kanamycin or 50 µg/mL ampicillin) for 12 h at 37°C, 250 rpm. Then the seed were transferred into 600 mL LB media with antibiotics and incubated at 37°C, 250 rpm to get OD_600_ of 0.8∼1.0. The recombinant protein production was initiated by adding isopropyl‐β‐d‐thiogalactoside (IPTG, final concentration of 0.5 mM) and cultures were grown at 16°C, 150 rpm for 20 h.

The *E. coli* cells were finally harvested by centrifugation (10,000 × g, 4°C, 20 min) and suspended in lysis buffer (20 mM Tris‐HCl, 0.5 M NaCl, pH 7.65). Cells were disrupted by high pressure homogenization (ATS‐1500, 4°C, 800 bar, 2 cycles) and the final lysate clarified by centrifugation at 12,000 g, 4°C for 30 min. The soluble fraction was subjected to 5 mL HisTrapTM HP column (Loaded onto ÄKTA Pure, Cytiva) and washed by washing buffer A (20 mM Tris‐HCl, 0.5 m NaCl, 10 mM imidazole, pH 7.65) and washing buffer B (20 mM Tris‐HCl, 0.5 M NaCl, 60 mM imidazole, pH 7.65), respectively. The target protein was eluted with an elution buffer (20 mM Tris‐HCl, 0.5 m NaCl, 300 mM imidazole, pH 7.65). Afterward, the buffer was exchanged for a storage buffer (20 mM Tris‐HCl, 0.5 m NaCl, pH 7.65) by using suitable ultrafiltration devices at 5,500× g, 4°C and glycerol (final concentration of 20%, v/v) was added in the end. The Protein purity was confirmed by SDS‐PAGE and concentration was determined by Pierce BCA Protein Assay Kits with bovine serum albumin as reference. The purified enzyme was stored at ‐80°C for further utilization.

### 4.8 Kinetic Analysis

To validate if the new candidates were well expressed, the activity was determined by 4‐Nitrophenol butyrate (*p*NPB, model substrate). All reactions were performed in a 96‐well plate, the final concentrations of *p*NPB range from 0.2 to 1.4 mM and final concentration of enzyme was 50 µg/mL. 10 µL of solution of *p*NPB (dissolved in anhydrous ethanol), 10 µL of enzymes and 80 µL reaction buffer (100 mM KH_2_PO_4_‐NaOH, pH 8.5) were incubated at 65°C for 3 min. Then, an equal volume of ethanol was added to quench the reaction. The products were measured at 405 nm using the UV–Vis plate reader (Bio‐Tek H1). Each reaction was performed in triplicate, the activity was calculated by Lineweaver‐Burk method.

### 4.9 Effects of Different pH and Temperature on Candidates

Enzymatic pH dependency was systematically evaluated under thermostatic conditions (65°C) using KH_2_PO_4_‐NaOH (100 mM) containing pH range of 7.0 to 9.0, with the final *p*NPB concentration of 1 mM. Temperature optima were resolved using pre‐equilibrated reactions (30°C–70°C, 10°C intervals) in 100 mM KH_2_PO_4_‐NaOH (pH 8.5) containing 1 mM *p*NPB. All assays employed triplicate technical replicates in 200 µL reaction volumes, terminated by ethanol addition (1:1 v/v). The products were measured at 405 nm by plate reader, and the relative activity was calculated by equation:

$$Relative\;activity=\frac{OD_{test}-OD_{blank}}{{\left(OD_{test}-OD_{blank}\right)}_{Best}}\times 100\%$$

where, *OD_test_* represents the absorbance of each reaction and *OD_blank_* represents the absorbance of corresponding condition.

### 4.10 Preparation of PET MPs and Fluorescent PET MPs

Two distinct PET MPs types were synthesized from semi‐crystalline (CryPET, GoodFellow ES30‐PD‐000132) and post‐consumer PET to enable mutant library screening and application assessment. The preparation followed established protocols with minor modifications [22, 74]. As shown in Figure S10, 1 g PET fragments were dissolved in 10 mL 90% trifluoroacetic acid (TFA, (v/v) TFA/ H2O) and stirred at 50°C for 2 h. The solution was then diluted with 10 mL 20% trifluoroacetic acid and continuously stirred for 24 h. Centrifugation at 2,500 × g for 1 h separated the supernatant from the sediment pellet. The pellet was subsequently resuspended in 100 mL 0.5% SDS buffer, followed by ultrasonic dispersion. Finally, PET MPs were filtered through a 150 µm filter and buffer‐exchanged for downstream applications (shown as Figure S15). Notably, the TFA treatment could reduce crystallinity (physical change) and induces minor terminal end‐capping (chemical change at the extremities), but it preserves the integrity of the main‐chain ester bonds. The resulting particles remain authentically high‐molecular‐weight PET (see Note S2 for details) [75, 76, 77].

The fluorescent PET MPs were prepared followed previous report with minor changes [78]. Briefly, Nile Red (Sigma‐Aldrich, suitable for fluorescence, Bioreagent, ≥97.0%) was dissolved in acetone (1 mg/mL) and added to 5 mg of Pc‐PET MPs. The mixture was stirred for 2 h, incubated at 4°C for 24 h, then centrifuged at 4000 g for 10 min. Pellets were washed sequentially with acetone and PBS, and stored at room temperature in the dark until use.

### 4.11 Depolymerization of Commercial GoodFellow PET and Pc‐PET MPs

To validate enzymatic depolymerization capacity of novel candidates, we established a dual‐substrate screening system utilizing semi‐crystalline PET powder (CryPET; GoodFellow ES30‐PD‐000132) and post‐consumer PET. The AhPETase mutant library was screened using CryPET MPs as substrate, while post‐consumer PET MPs (Pc‐PET MPs) assessed real‐scenario performance. Prepared substrates (10 mg) were incubated with 10 µM purified enzyme in 500 µL KH_2_PO_4_‐NaOH buffer (100 mM, pH 8.5) within 96‐deep‐well plates. Enzymatic digestion proceeded under controlled conditions (37°C, 900 rpm orbital agitation) for 96 h in a temperature‐regulated microplate shaker (Multitron, INFORS). To strictly eliminate batch‐to‐batch technical variations, the Wild‐Type AhPETase was included as an internal baseline control in every independent experimental assay. Reactions were performed in triplicate and quenched with 500 µL methanol, followed by centrifugation (4,000 × g, 20 min, 4°C) to isolate soluble products.

Following the enzymatic degradation assay, the remaining Pc‐PET MPs were collected, and the supernatant was completely removed. Crucially, the solid pellet was resuspended and vigorously washed with double‐distilled water, followed by centrifugation. This washing cycle was repeated twice to thoroughly remove any non‐specifically adsorbed proteins and buffer salts before the final drying and weighing steps.

### 4.12 Cell Culture

The human colon adenocarcinoma Caco‐2 cell line was cultured in flasks using Gibco BASIC MEM medium supplemented with 20% fetal bovine serum (Gibco Fetal Bovine Serum, Premium Plus) and 1% penicillin‐streptomycin (Gibco Penicillin‐Streptomycin (10,000 U/mL)). The BEAS‐2B cell line, derived from human bronchial epithelium, was cultured in DMEM medium (Gibco DMEM, high glucose, GlutaMAX Supplement) containing 10% fetal bovine serum and 1% penicillin‐streptomycin. Both cell lines were maintained in a humidified incubator at 37°C with 5% CO_2_. When the cells reached 80–90% of flakes, they were washed with 50 mM HEPES (Gibco HEPES (1 M)), digested (Gibco Trypsin‐EDTA (0.25%), phenol red), resuspended, and diluted in the respective culture media for passaging. Cells were passaged weekly until they reached a stable state suitable for testing.

### 4.13 Cytotoxicity Evaluation of Pc‐PET MPs and Degradation Products

The prepared Pc‐PET MPs were soaked in 75% ethanol overnight to fully sterilize, washed by PBS and resuspended different cell culture media at concentration of 10, 50, 100, 500, 1,000 µg/mL. The concentration gradient was carefully established based on a combination of recent toxicological consensus, physiological accumulation scenarios [20, 22] (see Note S1 for more details). The main degradation products including ethylene glycol, terephthalic acid and mono(2‐hydroxyethyl) terephthalate (MHET) were dissolved in corresponding culture media and sterilized by 0.22 µm filter membrane. The concentrations of each product were 10, 100, 500, 1,000, 5,000 µg/mL. In the Pc‐PET MPs and AhPETase^M1^ co‐treatment experiment, 1,000 µg/mL Pc‐PET MPs and 10 µM enzyme were configured in different culture media for further incubation. The AhPETase^M1^ used in heat‐inactivated enzyme control group was incubated at 95°C for 60 min, and complete loss of catalytic function was strictly verified using the *p*NPB assay prior to co‐treatment with cells.

Two cell lines were seeded in 96‐well plates at a density of 5,000 cells/well. After the cells were completely adhered, the experimental group, control group and blank were set in triplicate with 48 h treatment. Finally, the incubation supernatant was removed, 150 µL CCK‐8 working solution (90% culture media and 10% CCK‐8 solution) was added and incubated for 2 h before measuring the absorbance in UV–Vis plate reader (Bio‐Tek H1) at 450 nm. The cell viability was calculated as follows:

$$Cell\;viability=\frac{OD_{Experimentgroup}-OD_{Blank}}{OD_{Control}-OD_{Blank}}\times 100\%$$

### 4.14 Confocal Microscopy Assessment of Fluorescent Pc‐PET MP Internalization

Two cell lines were seeded in 35 mm glass‐bottom confocal dishes at 1 × 10^5^ cells/well and incubated until full adhesion. Culture media were replaced with 100 µg/mL fluorescent Pc‐PET MPs in fresh media. After 48 h exposure, cells were fixed with 4% paraformaldehyde for 10 min. After aspirating the paraformaldehyde, actin tracker green work solution (2% actin green, 2.5% BSA and 0.1% Triton X‐100, v/v) was added and incubated for 30 min. Then, washed the cells twice by PBS (0.1% Triton X‐100 added) and counterstained with DAPI. All procedures were performed under light‐protected conditions. Imaging used a Nikon A1 HD25 confocal microscope with excitation wavelength settings: 561 nm (Fluorescent Pc‐PET MPs), 488 nm (Actin Tracker), and 405 nm (DAPI).

#### 4.14.1 Scanning Electron Microscope (SEM)

PET MPs were mounted onto carbon tape and sputter‐coated with a 5 nm layer of gold using argon plasma. Scanning electron microscopy imaging was performed using a Zeiss Gemini450 field‐emission scanning electron microscope. The images were acquired at an accelerating voltage of 1.00/2.00 kV.

### 4.15 Transcriptomic and Proteomic Integrated Analysis

Samples were prepared as three groups: controls (routine culture media), Plasonly (control group supplemented with 1,000 µg/mL Pc‐PET MPs), and PlasEnzy (plastic‐only group supplemented with 10 µM sterile‐filtered AhPETase^M1^), with each group prepared in triplicate. Cells were harvested for proteomic analysis, and total RNA was extracted using TRIzol Reagent followed by immediate freezing in liquid nitrogen. Transcriptomic and proteomic integrated analyses were conducted by Shanghai Majorbio Bio‐pharm Biotechnology Co., Ltd.

RNA purification, reverse transcription, library construction, and sequencing followed the manufacturer's protocols. For transcriptomic analysis, the raw paired‐end reads were trimmed and quality‐controlled by fastp with default parameters [79]. The clean reads were then separately aligned to the Homo sapiens reference genome (GRCh38, Ensembl) with orientation mode using HISAT2 software [80]. The mapping rates across all samples were highly robust, ranging from 93.67% to 97.61%. Subsequently, the mapped reads of each sample were assembled by StringTie in a reference‐based approach [81]. Differentially expressed genes (DEGs) were identified by calculating expression levels using the transcripts per million (TPM) method. Gene abundance was quantified with RSEM, and differential expression analysis used DESeq2. Gene Ontology (GO) and KEGG pathway analyses identified significantly enriched terms and pathways among DEGs (Benjamini‐Hochberg‐corrected Padjust < 0.05 against the whole‐transcriptome background).

Total protein extraction, protein digestion, quantification, DIA mass spectrometry detection, and protein identification followed the manufacturer's protocols. P‐values and fold changes (FC) for proteins between groups were calculated using the R package “t‐test.” Differentially expressed proteins (DEPs) were identified using thresholds of |FC| > 1.2 and p < 0.05. Functional annotation of all identified proteins was performed using GO and KEGG pathway databases. DEPs were further subjected to GO and KEGG

### 4.16 UPLC Analysis of Degradation Products

Quantification was performed on a Waters ACQUITY Premier ultra‐performance liquid chromatography (UPLC) system equipped with a photodiode array detector (PDA). The analysis utilized an ACQUITY Premier BEH C18 Column (130Å pore size, 1.7 µm particle size, 2.1 × 50 mm; Waters) maintained at 40°C, with detection wavelength set to 260 nm. The mobile phase consisted of (A) 0.1% (v/v) formic acid in deionized water and (B) acetonitrile, delivered at a constant flow rate of 0.4 mL/min. A multistep gradient elution protocol was employed: initial 20% B, linear increase to 50% B over 1 min to separate TPA and MHET, followed by a rapid ramp to 70% B within 20 s and kept for 2 min for separation of larger products and contaminants. The system returned to initial conditions (20% B) at 3 min 20s and maintained for 1 min 40s to ensure column re‐equilibration, achieving a total run time of 5 min. According to the previous research, the activity was quantified by TPA and MHET released [29, 30, 31, 32, 35] and the peak details for UPLC quantification were shown in Note S3. Peaks were identified by comparing with high‐purity TPA and MHET standards and quantified using Waters Empower3 software‐integrated peak areas.

### 4.17 Statistical Analysis and Bioinformatics Programs

All experimental data are expressed as the mean ± standard deviation (s.d.) from three independent biological replicates (n = 3). Statistical evaluations were performed using IBM SPSS Statistics 24. Prior to comparative analysis, the normality of data distribution and the homogeneity of variances were rigorously assessed using the Shapiro‐Wilk test (corroborated by P‐P plots) and Levene's test, respectively. For datasets exhibiting normal distribution and equal variances (Levene's test *P*>0.05), a standard One‐Way Analysis of Variance (ANOVA) followed by Tukey's post hoc test was employed. For data where the assumption of equal variances was violated (Levene's test *P*<0.05), Welch's ANOVA followed by Dunnett's T3 post hoc test was utilized to ensure robustness. Statistical significance was defined as *∗P*<0.05. More specific details in statistical analysis were shown in Note S7, the parameters used for bioinformatics programs were shown in Table S6 and Note S8.

## Author Contributions

This project was conceived by Yajie Wang and Yuxuan Wang. The study design was developed by Yuxuan Wang and Yajie Wang. Yuxuan Wang was responsible for the collection of PETase dataset, model training and analyzing, boltz‐2 docking, enzyme library preparation, expression and purification, substrate preparation, functional testing, and cell experiments. Shijie He, Yuheng Chang and Sheng Mao assisted in enzyme expression, purification, and functional testing. Jianan Canal Li assisted in deep learning model development and explanation. Mingchun Xu and Chenxu Liu contributed to part of mutagenesis library screening. Binbin Chen and Hongxun Gao were involved in molecular docking, results analysis, and review. The manuscript was collaboratively written and has been reviewed by Yuxuan Wang and Yajie Wang.

## Conflicts of Interest

The authors declare no conflict of interest.

## Supporting information




**Supporting File**: advs77163‐sup‐0001‐SuppMat.docx.

## Data Availability

The data that support the findings of this study are available from the corresponding author upon reasonable request.
