# Supplementary material for: Deep Learning‐Driven Discovery and Engineering of an Efficient PETase for Depolymerization and Detoxification of PET Microplastics Under Physiological Conditions
Source: Adv Sci (Weinh). 2026 Aug 18;13(62):e77163. doi: 10.1002/advs.77163 (PMC13483144; doi:10.1002/advs.77163)
Supplement: Supplementary file 1 [file ADVS-13-e77163-s001.docx]

**Deep Learning-Driven Discovery and Engineering of an Efficient PETase for Depolymerization and Detoxification of PET Microplastics under Physiological Conditions**

Yuxuan Wang^1,2,3^, Shijie He^4,7^, Yuheng Chang^4,7^, Sheng Mao^4,7^, Jianan Canal Li^2^, Binbin Chen^2,3^, Hongxun Gao^3,4^, Mingchun Xu^4,7^, Chenxu Liu^4,7^, Yajie Wang^2,3,4,5,6^*

1. College of Environmental and Resource Sciences, Zhejiang University, Hangzhou 310058, China

2. School of Engineering, Westlake University, Hangzhou 310014, Zhejiang, China

3. The Center for Synthetic Biology and Integrated Bioengineering, Westlake University, Hangzhou 310014, Zhejiang, China

4. School of Life Science, Westlake University, Hangzhou 310014, Zhejiang, China

5. Westlake Laboratory of Life Sciences and Biomedicine, Xihu District, Hangzhou 310024, Zhejiang Province, China

6. Muyuan laboratory, Zhengzhou, Henan, China

7. Center for General Education, Westlake University, Hangzhou 310024, China

*Corresponding author. Email: wangyajie@westlake.edu.cn

**Table of contents**

**Supplementary Figures**

Figure S1 t-SNE visualization of embeddings before and after fine-tuning.

Figure S2 Identification overlaps of novel PETase candidates using distinct query strategies.

Figure S3 SDS-PAGE analysis of query proteins and soluble-expression candidates.

Figure S4 Structural and functional characterization of 20 novel PETase candidates.

Figure S5 Sequence-structure divergence of novel PETase candidates versus queries.

Figure S6 Amino-acid sequence alignment of different PET hydrolases.

Figure S7 Structural depiction of the interactions mediated by Leu61 in AhPETase.

Figure S8. Evolution of PET hydrolysis product profiles during AhPETase^M1^ engineering.

Figure S9. Sustained PET hydrolysis by AhPETase^M1^ over 21 days.

Figure S10 Synthesis workflow of post-consumer PET microplastics (Pc-PET MPs).

Figure S11 Quantification of PET depolymerization products by AhPETase^M1^ in MEM media

Figure S12 Bright-field microscopy of BEAS-2B and Caco-2 exposed to Pc-PET MPs.

Figure S13 Transcriptomic and proteomic integrated analysis.

Figure S14 SDS-PAGE and UPLC analysis of TrxA-SUMO dual tag strategy.

Figure S15 Morphological characterization of synthesized Pc-PET MPs.

Figure S16 DSC analysis results of four post-consumer PET materials and Pc-PET MPs.

**Supplementary Tables**

Table S1 The five-fold evaluation results of the fine-tuned PETase searching model.

Table S2 Wetlab-based initial screening of PETase candidates via kinetic parameters to pattern substrate 4-Nitrophenyl butyrate.

Table S3 PETase and newly mined candidates used in this study.

Table S4 Sequences of all the enzymes used in this study.

Table S5 Primers used in library construction.

Table S6 Summary of Bioinformatics and Computational Tools.

Table S7 Quantification of Pc-PET MPs degradation products under actual 48 h cell exposure conditions

Table S8 Crystallinity of four raw PET materials and Pc-PET MPs

**Supplementary Notes**

Supplementary Note 1 Criteria for establishing the concentration gradient of PET MPs to assess Pc-PET MP toxicity and the potential detoxifying effect of AhPETase^M1^.

Supplementary Note 2 An in-depth explanation of the physicochemical alterations to PET following TFA treatment, specifically focusing on changes in crystallinity and ester bond integrity.

Supplementary Note 3 UPLC details for the products quantification criteria.

Supplementary Note 4 Details of approaches, configuration, and iterations of the fine-tuning process.

Supplementary Note 5 Strategy for normalizing the model to prevent overfitting.

Supplementary Note 6 Significance of the selected ML model for zero-shot protein fitness prediction.

Supplementary Note 7 Statistical analysis in Figure 4d, 4e and 4f.

Supplementary Note 8 Parameters and criteria used for the bioinformatics programs.

**Figure S1 t-SNE visualization of embeddings before and after fine-tuning.**

The embeddings of positive and negative PETases before and after fine-tuning. t-SNE visualization reveals distinct separation between PETase (positive) and non-PETase (negative) clusters in ESM-1b embedding space, demonstrating enhanced discrimination after fine-tuning.

**Figure S2 Identification overlaps of novel PETase candidates using distinct query strategies.**

Venn diagram illustrating candidate distribution identified through independent search approaches. Overlapping regions denote shared candidates exhibiting higher confidence as functional PETases due to consistent detection across methodologies.

**Figure S3 SDS-PAGE analysis of query proteins and soluble-expression candidates.**

Demonstrates purity and molecular weight of purified proteins across independent fermentation batches.

**Figure S4 Structural and functional characterization of 20 novel PETase candidates.**

Predicted tertiary structures generated using AlphaFold 2.2. Enzymatic activity screening: Recombinant proteins were expressed in *E. coli* C41(DE3) using pET-21b (+) plasmids, with activity assessed against 4-nitrophenyl butyrate (4-NPB) as a model substrate.

**Figure S5 Sequence-structure divergence of novel PETase candidates versus queries.**

Novel candidates exhibit low sequence similarity (mean 42.37%, range: 30.04–47.25%, blue) but high structural conservation (mean TM-score 0.91, range: 0.77–0.96, red) relative to five reference PETases.

**Figure S6 Amino-acid sequence alignment of different PET hydrolases.**

Alignment of AhPETase, MG8 and five queries are compared. Secondary structure elements are drawn based on the AlphaFold structure of AhPETase and shown with black helices (α-helix) and arrows (β-sheet). The conserved serine hydrolase Gly-x1-Ser-x2-Gly motif, disulfide bond and extended loop are highlighted as purple, light orange and red, respectively. Residues involved in catalytic triad are shown in pink triangle. The putative substrate binding sites of AhPETase are presented as green circles. Similar with other lipases and esterases with PET hydrolysis capabilities, the structure of AhPETase retained the conserved serine hydrolase Gly-x1-Ser-x2-Gly motif (Gly127-His128-Ser129-Met130-Gly131), and the central twisted β-sheet is formed by nine mixed β-strands (β1-β9) and embraced by six α-helices (α1-α6) and five 3_10_-helices (η1-η5).

**Figure S7 Structural depiction of the interactions mediated by Leu61 in AhPETase.**

Distances (in Ångströms) to key neighboring residues are indicated in yellow dash line.

**Figure S8 Evolution of PET hydrolysis product profiles during AhPETase^M1^ engineering.**

UPLC-quantified release of MHET and mono(2-hydroxyethyl) terephthalate (MHET) and terephthalic acid (TPA) across enzyme variants. Key performance-altering mutants are highlighted (as indicated).

**Figure S9 Sustained PET hydrolysis by AhPETase^M1^ over 21 days.**

Time-resolved UPLC quantification of terephthalic acid (TPA) and mono(2-hydroxyethyl) terephthalate (MHET) release. Progressive decline in catalytic activity occurred with substrate accumulation, though PET degradation persisted through day 21.

**Figure S10 Synthesis workflow of post-consumer PET microplastics (Pc-PET MPs).**

Four post-consumer PET sources were fragmented, dissolved in 90% (v/v) trifluoroacetic acid (TFA)/H_2_O, precipitated with 20% (v/v) TFA/ H_2_O, filtered (150 μm), and resuspended in specified buffer for further application.

**Figure S11 Quantification of PET depolymerization products by AhPETase^M1^ in MEM media.**

Quantification of PET depolymerization products (TPA and MHET) by AhPETase^M1^. The assay was conducted using 10μM enzyme and10mg post-consumer PET MPs (Pc-PET MPs) directly in complex MEM basal cell culture media (~ pH 7.4) at 37 °C during 7-day period. The continuous accumulation of TPA (black solid line) and the progressive consumption of the MHET intermediate (blue dashed line) confirm the robust catalytic viability and stability of AhPETase^M1^ in a true physiological environment. Data are presented as mean ± s.d. (n=5 independent replicates).

To strictly align with the core theme of our study, depolymerization under physiological conditions, we performed comprehensive new PET depolymerization assays under stringent physiological parameters:

1. Depolymerization at physiological pH (~7.4) in complex media

We utilized a freshly purified batch of AhPETase^M1^ to conduct a 7-day depolymerization assay of post-consumer PET MPs (Pc-PET MPs) directly in MEM basal cell culture media (~pH 7.4) at 37 °C.

2. Simulation of physiological shear stress (mechanical agitation)

Crucially, to accurately simulate the dynamic physical environment of human biological systems (such as gastrointestinal peristalsis or circulatory hemodynamics), these neutral-pH assays were conducted in a microplate shaker maintained at a high orbital agitation speed of 900 rpm. This mechanical parameter reflects the dynamic *in vivo* environment where MPs and enzymes interact to some extent.

As shown in Figure S11, AhPETase^M1^ exhibited remarkable and sustained depolymerization activity under these stringent physiological conditions (MEM, ~pH 7.4, 37 °C, 900 rpm).

**Figure S12 Bright-field microscopy of BEAS-2B and Caco-2 exposed to Pc-PET MPs.**

Microplastics aggregation becomes visible at concentrations >50 μg/mL.

**Figure S13 Transcriptomic and proteomic integrated analysis.**

**a** Volcano plot depicting differential gene expression in BEAS-2B cells, with the log_2_(fold change) on the x-axis and -log_10_(*P*_adjust_) on the y-axis. Smaller *P*_adjust_ values denote greater statistical significance. **b** Volcano plot depicting differential gene expression in Caco-2 cells. Analysis methodology matches Fig. A. **c** KEGG functional annotation analysis comparing PlasEnzy-treated and control BEAS-2B cells. Secondary KEGG pathway categories are shown on the y-axis, while the x-axis represents the number of annotated genes per pathway. **d** KEGG pathway enrichment analysis for differentially expressed genes in BEAS-2B cells. Point size indicates the number of genes within the pathway, and a larger Rich factor signifies a greater degree of enrichment. **e** Multi-group differential analysis of proteins in BEAS-2B cells. Protein names are shown on the y-axis. The x-axis represents mean relative expression across groups, with bar colors denoting group assignments. Significance levels: **P* ≤ 0.05; ***P* ≤ 0.01; ****P* ≤ 0.001 (one-way ANOVA with Tukey's post hoc test). **f** Functional annotation analysis of GO terms for PlasEnzy-treated versus control Caco-2 cells. The y-axis displays secondary GO categories, while the x-axis indicates the number of genes annotated to each category. Colors represent the three primary GO classifications: biological process (BP), cellular component (CC), and molecular function (MF). **g** Multi-group differential analysis of proteins in Caco-2 cells. Analysis methodology matches Figure S13e.

**Figure S14 SDS-PAGE and UPLC analysis of TrxA-SUMO dual tag strategy. Solubility enhancement of MG8 through TrxA-SUMO fusion tagging. (Top, left) SDS-PAGE analysis of purified TrxA-SUMO AhPETase and AhPETase used for validation, confirming its purity and expected molecular weight (Top, right). Products quantification by UPLC to compare the activity between AhPETase and TrxA-SUMO tagged AhPETase (Bottom, data are presented as mean ± s.d. n=3).**

SDS-PAGE analysis comparing expression profiles: Lanes 1-3 were expressed by pET-21b (+): 1) Whole-cell lysate, 2) Insoluble fraction, 3) Purified insoluble MG8; Lane 4: Purified soluble TrxA-SUMO-MG8 fusion from pET-32a (+). The MG8 sequence was subcloned via Gibson assembly (primers in Table S5) to enable soluble expression.

To determine if this massive fusion tag artificially disadvantaged MG8 in our activity comparisons (due to potential steric hindrance), we performed a rigorous control experiment during this revision. We engineered a new construct where our AhPETase was fused with the exact same dual TrxA-SUMO tag. We then expressed, purified, and compared its catalytic activity against our original, lightly-tagged AhPETase.

Our empirical assays revealed that the TrxA-SUMO fusion tag actually enhanced, rather than hindered, the overall catalytic performance of AhPETase (2.44 ± 0.50 fold improvement). Consequently, our original comparison, evaluating our AhPETase^M1^ against the TrxA-SUMO tagged MG8, did not artificially disadvantage MG8. Therefore, our claim of a "34-fold improvement" represents a conservative underestimate of AhPETase^M1^'s relative superiority.

**Figure S15 Morphological characterization of synthesized Pc-PET MPs.**

Scanning electron microscopy (SEM) reveals a polydisperse size distribution ranging from sub-micrometer to multi-micrometer scales.

**Figure S16 DSC analysis results of four post-consumer PET materials and Pc-PET MPs.**

**Table S1 The five-fold evaluation results of the fine-tuned PETase searching model**

**Table S2 Wetlab-based initial screening of PETase candidates via kinetic parameters to pattern substrate 4-Nitrophenyl butyrate (*p*NPB)**

**Table S3 PETase and newly mined candidates used in this study**

| **Entry** | **Name** | **UniProt ID** | **Organism** | **Status** | **History*** |
| --- | --- | --- | --- | --- | --- |
| 1 | IsPETase | A0A0K8P6T7 | *Ideonella sakaiensis* | Reviewed | 11-Nov-2015 |
| 2 | LCC | G9BY57 | *Unknown prokaryote* | Reviewed | 22-Feb-2012 |
| 3 | BhrPETase | A0A2H5Z9R5 | *bacterium HR29* | Unreviewed | 28-Feb-2018 |
| 4 | Cut190 | C7MVE8 | *Saccharomonospora viridis* | Unreviewed | 13-Oct-2009 |
| 5 | TfCut2 | E9LVH8 | *Thermobifida cellulosilytica* | Reviewed | 05-Apr-2011 |
| 6 | N/A | A0A317D8W8 | *Micromonospora acroterricola* | Unreviewed | 10-Oct-2018 |
| 7 | N/A | A0A3A3YUA9 | *Vallicoccus soli* | Unreviewed | 05-Dec-2018 |
| 8 | N/A | P0DX29 | *Amycolatopsis mediterranei* | Reviewed | 13-Sep-2023 |
| 9 | Candidate 015 | A0A431KNH0 | *Burkholderiales bacterium* | Unreviewed | 08-May-2019 |
| 10 | N/A | A0A5Q2RDB8 | *Actinomarinicola tropica* | Unreviewed | 26-Feb-2020 |
| 11 | N/A | A0A2D5A8E5 | *Planctomycetaceae bacterium* | Unreviewed | 25-Apr-2018 |
| 12 | N/A | A0A225C6B8 | *Clavibacter michiganensis* subsp. *tessellarius* | Unreviewed | 25-Oct-2017 |
| 13 | N/A | A0A4Q7L6Z1 | *Herbihabitans rhizosphaerae* | Unreviewed | 31-Jul-2019 |
| 14 | N/A | G2NZQ2 | *Streptomyces violaceusniger* | Unreviewed | 16-Nov-2011 |
| 15 | N/A | A0A285KUL5 | *Actinoplanes atraurantiacus* | Unreviewed | 22-Nov-2017 |
| 16 | N/A | A0A1H5UML9 | *Nonomuraea solani* | Unreviewed | 18-Jan-2017 |
| 17 | N/A | A0A9D8JHD9 | *Deltaproteobacteria bacterium* | Unreviewed | 03-May-2023 |
| 18 | N/A | K6W498 | *Austwickia chelonae NBRC 105200* | Unreviewed | 09-Jan-2013 |
| 19 | N/A | A0A540Q4K0 | *Streptomyces ipomoeae* | Unreviewed | 16-Oct-2019 |
| 20 | N/A | UPI001C5A3EA1 | *Austwickia* sp. *TVS 96-490-7B* | Unreviewed | N/A |
| 21 | N/A | A0A2T5PA95 | *Pseudomonas mangrovi* | Unreviewed | 18-Jul-2018 |
| 22 | N/A | UPI0015F00EDD | *Streptomyces phytophilus* | Unreviewed | N/A |
| 23 | N/A | A0A2D9H8X5 | *Myxococcales bacterium* | Unreviewed | 25-Apr-2018 |
| 24 | N/A | F7IX06 | *Thermobifida alba* | Reviewed | 21-Sep-2011 |
| 25 | N/A | A0A1S8XX03 | *Micromonospora* sp*. Rc5* | Unreviewed | 10-May-2017 |
| 26 | N/A | U7MPV8 | *Corynebacterium* sp. *KPL1989* | Unreviewed | 22-Jan-2014 |
| 27 | N/A | A0A3M8K6J4 | *Corynebacterium alimapuense* | Unreviewed | 13-Feb-2019 |
| 28 | N/A | UPI002109C9E1 | *Streptomyces* sp. *ODS25* | Unreviewed | N/A |
| 29 | N/A | UPI00122DE792 | *Streptomyces tailanensis* | Unreviewed | N/A |
| 30 | N/A | A0A2D5S4U2 | *Planctomycetaceae bacterium* | Unreviewed | 25-Apr-2018 |
| 31 | N/A | UPI0015888D6E | *unclassified Burkholderia* | Unreviewed | N/A |
| 32 | N/A | A0A0D8HR84 | *Rhodococcus* sp. | Unreviewed | 27-May-2015 |
| 33 | N/A | A0A1H6AD45 | *Halopseudomonas aestusnigri* | Unreviewed | 18-Jan-2017 |
| 34 | N/A | A0A1N6M769 | *Vibrio spartinae* | Unreviewed | 15-Mar-2017 |
| 35 | N/A | A0A117E7J1 | *Streptomyces acidiscabies* | Unreviewed | 13-Apr-2016 |
| 36 | N/A | A0A918GHB6 | *Actinokineospora fastidiosa* | Unreviewed | 22-Feb-2023 |
| 37 | N/A | UPI0005658317 | *Streptomyces* sp. *NRRL S-475* | Unreviewed | 27-Mar-2024 |
| 38 | N/A | A0A9E3E783 | *Streptosporangiaceae bacterium* | Unreviewed | N/A |
| 39 | N/A | A0A956SY07 | *Myxococcales bacterium* | Unreviewed | 22-Feb-2023 |
| 40 | Candidate 007 | A0A7W7MJQ0 | *Actinoplanes lobatus* | Unreviewed | 02-Jun-2021 |
| 41 | N/A | B0RFW0 | *Clavibacter sepedonicus* | Unreviewed | 08-Apr-2008 |
| 42 | N/A | A0A4U6QFA3 | *Nakamurella flava* | Unreviewed | 31-Jul-2019 |
| 43 | N/A | UPI000A4FA727 | *Streptomyces* sp. *SBT349* | Unreviewed | N/A |
| 44 | Candidate 016 | UPI00194F5999 | *Microbispora corallina* | Unreviewed | N/A |
| 45 | Candidate 009 | A0A0A0DZ00 | *Aquabacterium* sp. *NJ1* | Unreviewed | 07-Jan-2015 |
| 46 | N/A | A0A5P9PUN1 | *Amycolatopsis* sp. *YIM 10* | Unreviewed | 22-Apr-2020 |
| 47 | N/A | A0A2E1UB56 | *Phycisphaerae bacterium* | Unreviewed | 31-Jan-2018 |
| 48 | Candidate 019 | A0A6C1NB71 | *Halomonadaceae bacterium* | Unreviewed | 17-Jun-2020 |
| 49 | Candidate 017 | A0A7W9KQH4 | *Kutzneria kofuensis* | Unreviewed | 02-Jun-2021 |
| 50 | IsPETase | A0A0K8P6T7 | *Ideonella sakaiensis* | Reviewed | 11-Nov-2015 |
| 51 | N/A | A0A7W9KF39 | *Kutzneria kofuensis* | Unreviewed | 02-Jun-2021 |
| 52 | N/A | A0A1V2PVS9 | *Actinosynnema* sp. *ALI-1.44* | Unreviewed | 07-Jun-2017 |
| 53 | N/A | A0A973N794 | *Catenulispora* sp. *Bin4.6.1* | Unreviewed | 22-Feb-2023 |
| 54 | N/A | UPI000255D23B | *Nesterenkonia* sp. *F* | Unreviewed | N/A |
| 55 | N/A | A0A925R2S1 | *Rhizobacter* sp | Unreviewed | 22-Feb-2023 |
| 56 | N/A | UPI002362B8B6 | *Kutzneria chonburiensis* | Unreviewed | N/A |
| 57 | N/A | UPI00114CF723 | *Oligoflexus tunisiensis* | Unreviewed | N/A |
| 58 | N/A | A0A0S2KB11 | *Pseudohongiella spirulinae* | Unreviewed | 17-Feb-2016 |
| 59 | N/A | W7ISR7 | *Actinokineospora spheciospongiae* | Unreviewed | 16-Apr-2014 |
| 60 | N/A | A0A1H7PWV6 | *Roseateles* sp. *YR242* | Unreviewed | 22-Nov-2017 |
| 61 | N/A | A0A0A0DPV4 | *Aquabacterium* sp. *NJ1* | Unreviewed | 07-Jan-2015 |
| 62 | N/A | M5DT34 | *Thalassolituus oleivorans MIL-1* | Unreviewed | 29-May-2013 |
| 63 | AhPETase | A0A239PER3 | *Asanoa hainanensis* | Unreviewed | 25-Oct-2017 |
| 64 | N/A | A0A2E1M2I1 | *Gammaproteobacteria bacterium* | Unreviewed | 25-Apr-2018 |
| 65 | N/A | A0A4V6MTQ0 | *Aquabacterium lacunae* | Unreviewed | 31-Jul-2019 |
| 66 | N/A | A0A2P4UDA7 | *Actinomadura rubteroloni* | Unreviewed | 23-May-2018 |
| 67 | N/A | A0A381Z9M3 | *Marine metagenome* | Unreviewed | 07-Nov-2018 |
| 68 | Candidate 012 | A0A3R8T446 | *Aquabacterium soli* | Unreviewed | 10-Apr-2019 |
| 69 | N/A | A0A354TUM9 | *Gammaproteobacteria bacterium* | Unreviewed | 07-Nov-2018 |
| 70 | Candidate 014 | A0A940F8C9 | *Solirubrobacteraceae bacterium* | Unreviewed | 22-Feb-2023 |
| 71 | Candidate 005 | A0A1W6L7G8 | *Piscinibacter gummiphilus* | Unreviewed | 05-Jul-2017 |
| 72 | N/A | A0A7D3W2P3 | *Actinomadura verrucosospora* | Unreviewed | 10-Feb-2021 |
| 73 | N/A | UPI001438FF9C | *Neolewinella antarctica* | Unreviewed | N/A |
| 74 | Candidate 010 | A0A1W6LF85 | *Piscinibacter gummiphilus* | Unreviewed | 05-Jul-2017 |
| 75 | N/A | A0A2P4UNN9 | *Actinomadura rubteroloni* | Unreviewed | 23-May-2018 |
| 76 | Candidate 003 | A0A0U1PS69 | *Rathayibacter toxicus* | Unreviewed | 17-Feb-2016 |
| 77 | Candidate 013 | UPI002487CDC9 | *Aquabacterium* sp. | Unreviewed | N/A |
| 78 | N/A | UPI0025FD6B35 | *Rhodoferax* sp. | Unreviewed | N/A |
| 79 | N/A | A0A229TBU2 | *Amycolatopsis vastitatis* | Unreviewed | 25-Oct-2017 |
| 80 | N/A | A0A2M9BGS8 | *Mumia flava* | Unreviewed | 25-Apr-2018 |
| 81 | N/A | UPI0017494009 | *Amycolatopsis bullii* | Unreviewed | N/A |
| 82 | N/A | A0A8I1S4K5 | *Actinomycetes bacterium* | Unreviewed | 23-Feb-2022 |
| 83 | N/A | A0A078M5Q1 | *Pseudomonas saudimassiliensis* | Unreviewed | 29-Oct-2014 |
| 84 | N/A | A0A7X9JX82 | *Deltaproteobacteria bacterium* | Unreviewed | 02-Jun-2021 |
| 85 | N/A | A0A940JC07 | *Solirubrobacteraceae bacterium* | Unreviewed | 22-Feb-2023 |
| 86 | Candidate 002 | A0A0A0DQ06 | *Aquabacterium* sp. *NJ1* | Unreviewed | 07-Jan-2015 |
| 87 | N/A | UPI00237F945F | *Streptomyces* | Unreviewed | N/A |
| 88 | N/A | A0A3D9SRH8 | *Thermomonospora umbrina* | Unreviewed | 16-Jan-2019 |
| 89 | N/A | UPI000718ADAE | *Top level (root)* | Unreviewed | N/A |
| 90 | N/A | A0A939PI20 | *Actinomadura barringtoniae* | Unreviewed | 22-Feb-2023 |
| 91 | N/A | A0A2E0A2W8 | *Oceanospirillaceae bacterium* | Unreviewed | 31-Jan-2018 |
| 92 | N/A | A0A7X7EZ21 | *Bacteroidales bacterium* | Unreviewed | 02-Jun-2021 |
| 93 | N/A | A0A4P7XLW2 | *Hydrocarboniclastica marina* | Unreviewed | 31-Jul-2019 |
| 94 | N/A | X0RFS8 | *Psychrobacter* sp. *JCM 18903* | Unreviewed | 14-May-2014 |
| 95 | Candidate 020 | A0A0R2WZ58 | *Cryomorphaceae bacterium BACL22 MAG-120619-bin32* | Unreviewed | 20-Jan-2016 |
| 96 | N/A | A0A7W7RIU6 | *Lipingzhangella halophila* | Unreviewed | 02-Jun-2021 |
| 97 | Candidate 001 | A0A931F5V8 | *Nonomuraea cypriaca* | Unreviewed | 22-Feb-2023 |
| 98 | Candidate 008 | A0A2D4S776 | *Bermanella* sp. | Unreviewed | 25-Apr-2018 |
| 99 | N/A | UPI0023B24678 | *Actinoplanes tereljensis* | Unreviewed | N/A |
| 100 | Candidate 006 | A0A3D4HC96 | *Opitutae bacterium* | Unreviewed | 16-Jan-2019 |
| 101 | N/A | A0A2T5PBR4 | *Pseudomonas mangrovi* | Unreviewed | 18-Jul-2018 |
| 102 | N/A | UPI0021C3E726 | *Micromonosporaceae* | Unreviewed | N/A |
| 103 | Candidate 018 | UPI001CECAD91 | *Streptomyces* sp. *HPF1205* | Unreviewed | N/A |
| 104 | Candidate 004 | A0A395JIT5 | *Arenicella xantha* | Unreviewed | 05-Dec-2018 |

*: History refers to the first record of this sequence in UniProt dataset.

**Table S4 Sequences of all the enzymes used in this study**

IsPETase-pET-21b(+)

atgcaaacaaatccctatgctaggggacccaacccgaccgcggctagcttggaggcaagcgctggtccgtttaccgtgcgttcgttcaccgtcagccgtccgagcggttacggtgcgggtacggtctattacccgacgaatgctggcggtactgtgggcgccatcgcgattgttccgggttataccgcgagacaaagctccattaaatggtggggtccgcgtctggcaagccatggttttgtagttattaccattgataccaacagcactctggaccagccaagctcccgctcttctcagcaaatggctgcgctgcgtcaggttgcgagcttgaatggcacctccagttccccgatttacggcaaggtggacacggcccgtatgggcgtgatgggttggtcaatgggcggcggcggcagcctgatcagcgcggcgaacaacccgtcactgaaagcagccgcgccgcaagcaccgtgggatagctcgaccaacttcagttctgttaccgtgccgaccctgatcttcgcctgcgaaaacgacagcatcgcccctgttaattcctctgcattgccgatctacgacagtatgagccgcaacgctaaacagttcctggagatcaacggtggcagccacagctgtgcaaactccggtaatagcaatcaggcgctcatcggtaagaaaggtgtggcgtggatgaagcgctttatggataatgacacccgctatagcactttcgcgtgcgagaacccaaattctacgcgtgtttcggattttcgtaccgcgaactgcagccttgaactcgagcaccaccaccaccaccactga

MQTNPYARGPNPTAASLEASAGPFTVRSFTVSRPSGYGAGTVYYPTNAGGTVGAIAIVPGYTARQSSIKWWGPRLASHGFVVITIDTNSTLDQPSSRSSQQMAALRQVASLNGTSSSPIYGKVDTARMGVMGWSMGGGGSLISAANNPSLKAAAPQAPWDSSTNFSSVTVPTLIFACENDSIAPVNSSALPIYDSMSRNAKQFLEINGGSHSCANSGNSNQALIGKKGVAWMKRFMDNDTRYSTFACENPNSTRVSDFRTANCSLELEHHHHHH*

LCC-pET-26b(+)

atgtcaaatccttatcaaaggaaggacccaacccaaccccgccagcgcccttaccgcggacggcccgtttagcgtcgctacctatacctagaagcagattaagcgtggccggtttcggcggtggcgtgtgatctactacccgaccggtacgtcattgactttcggcggccatcgccatgagcccgggttacaccgcagacgcgagtagcttgcttgctgggtcgcgctggcgaccaaggctttgtgtctggtttattaataccaacaagccgtttcgacatatccggattctcgtgcttcccaactgtcggcggcgttaaattaatctgcgtaccagctcaccgtctgctgtgcgtgcctgttggccgattggacgctaatcgtctggcgtggcgggtcatagcatgggtggcggtgtgtaactctgcgtattgcggagcaaaacccgagcctgaaagcagcggttcccgctgaccccgtggcacaccgataagaccttcaacacgtccgtgccggttctcattgttggtgcagaggcggacacggtgtgcccagttagccaacatgctatcccgttttatcagaacctgtccgtcgaccaccccaaggtgtacgtggactggacaacgcctcccaactttgcaccgaattccaacaacgacggcgatctctgtctacacaattagctggatgaaactgtgggtgataacgatacccgttaccgccagtttcttgtgtaatgttaatgatccgccattgtctgatttccgcacgaacaaccgccattgccagctcgagcaccaccaccaccaccactga

MSNPYQRGPNPTRSALTADGPFSVATYTVSRLSVSGFGGGVIYYPTGTSLTFGGIAMSPGYTADASSLAWLGRRLASHGFVVLVINTNSRFDYPDSRASQLSAALNYLRTSSPSAVRARLDANRLAVAGHSMGGGGTLRIAEQNPSLKAAVPLTPWHTDKTFNTSVPVLIVGAEADTVAPVSQHAIPFYQNLPSTTPKVYVELDNASHFAPNSNNAAISVYTISWMKLWVDNDTRYRQFLCNVNDPALSDFRTNNRHCQLEHHHHHH*

BhrPETase-pET-21b(+)

atgtcaaatccttatcaaaggggacccaacccaaccccgctcggcgctgacgacggatggtcctttagcgtcgcgacctatagcgttagccgtttaagcgttagcggttttggcggtggcgtgtgatctactacccgaccggcaccaccctgacgttggcggccatcgctatgagcccgggttataccgcggacgcctcctcgccctggcggctgggtcgtcgtctcgcttcccatggttttgttattcgtcattcaacaccaatagccgtttggacttccctgtagagccgcggtccccaactgtctgctgcgctgaactacctgcgcacctcgtctccgagcgcggtcggtgcgcgtttggacgcaaatagattggcggtcggcaggccacagcatgggcggaggtcgactctgcgtatttccgagcaaattccgactctgaaagcaggtgtgccgctgaccccgtggcataccgacaagaccttcaacaccccaattccgcagcttatcgtgggtgcggaggccgacactgtaggcaccggttagccaacacgcaattccgttctatcagaacctgtccgtccaccacgccgaaggtgtacgtggattggacaacgccacccattttgcgccaaacagtccgaatgctgcgatcagcgtgtacacgattagttggatgaaattatgtggttgataacgacacccgttatcgccagtttcttgtgtaatgttaatgatccgccattgtctgatttccgcagcaacaaccgtcactgccagctgctcgagcaccaccaccaccaccactga

MSNPYQRGPNPTRSALTTDGPFSVATYSVSRLSVSGFGGGVIYYPTGTTLTFGGIAMSPGYTADASSLAWLGRRLASHGFVVIVINTNSRLDFPDSRASQLSAALNYLRTSSPSAVRARLDANRLAVAGHSMGGGATLRISEQIPTLKAGVPLTPWHTDKTFNTPVPQLIVGAEADTVAPVSQHAIPFYQNLPSTTPKVYVELDNATHFAPNSPNAAISVYTISWMKLWVDNDTRYRQFLCNVNDPALSDFRSNNRHCQLLEHHHHHH*

Cut190-pET-21b(+)

atgcaagataatccctatgaaaggggacccgacccaactgaggactccatcgaggctatcagaggtccgttcagcgttgccaccgaacgtgtttcttcttttgctagcggtttcggcggcggcaccatctattacccgcgtgaaaccgatgagggtacatttggtgcggtggctgtggcgcctggcttcaccgcatcgcaaggtagcatgagctggtatggtgagcgcgtcgcgtctcagggcttcatcgtttttaccatcgatacgaacacccgtctggatcagccgggtcaacgtggccgtcagttgctggcggctctggactacctggttgaacgcagcgatcgtaaagttcgcgagaggttggacccgaaccgtttggcggtcatgggccatagcatgggtggcggcggaagtttagaggcgactgtaatgcgtccgagccttaaggcgtcgattccgctgaccccgtggaatctggacaaaacctggggtcaggtgcaggttccgacgtttatcattggtgcggaattggacaccattgcgtccgtgcgtacccacgccaaaccgttctacgaatccttaccgtcctcactcccgaaagcatacatggaactggacggcgcgacccacttcgccccaaacattccgaacaccaccattgcaaagtacgtgatcagctggctgaagcgcttcgtggatgaagatacgcgttacagccaatttctgtgtccgaatccgacggaccgcgcaattgaggaatatcgtagcacctgcccgtatctggagctcgagcaccaccaccaccaccactga

MQDNPYERGPDPTEDSIEAIRGPFSVATERVSSFASGFGGGTIYYPRETDEGTFGAVAVAPGFTASQGSMSWYGERVASQGFIVFTIDTNTRLDQPGQRGRQLLAALDYLVERSDRKVRERLDPNRLAVMGHSMGGGGSLEATVMRPSLKASIPLTPWNLDKTWGQVQVPTFIIGAELDTIASVRTHAKPFYESLPSSLPKAYMELDGATHFAPNIPNTTIAKYVISWLKRFVDEDTRYSQFLCPNPTDRAIEEYRSTCPYLELEHHHHHH*

TfCu2-pET-21b(+)

atggctaatccttatgaaaggggacccaacccaacagatgcattgctggaggcgcgtagcggtccgttcagcgttagcgaagagaacgtttctcgcctctccgcgtctggcttcggcggcgggaccatctactacccgcgtgaaaataacacctatggtgcggtggccatctctccgggttatactggtactgaggcgagcattgcgtggctgggcgagcgcatcgccagtcatggtttcgtggttattaccattgacacgatcacgaccctcgaccagccggactctcgcgcagaacagctgaacgcagccttgaatcacatgattaaccgtgcgtcctccacggttcgtagccgcattgacagcagccgcctggcggtgatgggtcattccatgggcggtggcggttccctgcgtctggcaagccaacgtccggatttgaaggctgcgatcccgctgaccccgtggcatctgaacaagaactggagcagcgtcaccgtgccgaccctgattatcggtgctgatttggacacgatcgcgccagtggccacccacgcaaaaccgttctacaatagcttaccgagctcaatctccaaagcatatctggagctggacggcgctacccactttgcgccgaacatcccgaacaaaatcattggcaagtacagcgtcgcgtggctgaaacgttttgttgataatgatacccgttacacccaatttttgtgcccgggtccaagagatggcctgtttggtgaagttgaagaatatcgtagcacctgtcctttctacccgaacagcagctcggtggacaagctggcggctgcgttggagctcgagcaccaccaccaccaccactga

MANPYERGPNPTDALLEARSGPFSVSEENVSRLSASGFGGGTIYYPRENNTYGAVAISPGYTGTEASIAWLGERIASHGFVVITIDTITTLDQPDSRAEQLNAALNHMINRASSTVRSRIDSSRLAVMGHSMGGGGSLRLASQRPDLKAAIPLTPWHLNKNWSSVTVPTLIIGADLDTIAPVATHAKPFYNSLPSSISKAYLELDGATHFAPNIPNKIIGKYSVAWLKRFVDNDTRYTQFLCPGPRDGLFGEVEEYRSTCPFYPNSSSVDKLAAALELEHHHHHH*

MG8-pET-32a(+)

atgagcgataaaattattcacctgactgacgacagttttgacacggatgtactcaaagcggacggggcgatcctcgtcgatttctgggcagagtggtgcggtccgtgcaaaatgatcgccccgattctggatgaaatcgctgacgaatatcagggcaaactgaccgttgcaaaactgaacatcgatcaaaaccctggcactgcgccgaaatatggcatccgtggtatcccgactctgctgctgttcaaaaacggtgaagtggcggcaaccaaagtgggtgcactgtctaaaggtcagttgaaagagttcctcgacgctaacctggccggttctggttctggccatatgtcggactcagaagtcaatcaagaagctaagccagaggtcaagccagaagtcaagcctgagactcacatcaatttaaaggtgtccgatggatcttcagagatcttcttcaagatcaaaaagaccactcctttaagaaggctgatggaagcgttcgctaaaagacagggtaaggaaatggactccttaagattcttgtacgacggtattagaattcaagctgatcagacccctgaagatttggacatggaggataacgatattattgaggctcacagagaacagattggtgacgacgacgacaaggccatgaacaatccgcctcccccagatgaccccggagctccgtccccgtaccagcgtggtccggacccaacggtgagcttcgttgaagcttctcgcggcaactaccgtgttgcgaccagcaatgtctcgtcactggtttcaggcttcggcggtggtactattcattatccgtctaacgctaccggtacgatgggcgcgatcgtggtcattccgggttatgtttccggtgagggatccatcgactggtggggtccgaagctggcaagctatggtttcgtggtgatgaccatcggcaccaatagcggcttcgatcaaccaccgagccgggcacgtcagattaacaacgcattagactatctggtcgagcagaacacccgtaccggtagcccggttagaggcatgattgacccgagccgtttgggcgtgatcgggtggagcatgggtggtggtggcacgatccgtgtagccggtgagggccgcattaaggcagcgatcccgctggccccttgggatacctccagcatcccgtctcgcggcgtgcaggctccgaccctgatttttgcgtgccagtccgatgtggttgcgccggttcgtagccatgcgtctccgttttataacgcgctgccgggttcgatcagcaaagcctttgtgaatctcaataacggtaaccacttctgcgccaatggtggcagcagcttcggccgttacgacgcggcgttgggtcgtctgggtgttagctggatgaaacgctttttggacgaagatcgtcgttacagccaatttctgtgtggtcccaaccacaccggcgatcgccaaattaccgaataccgcggcaactgcccgtacctcgagcaccaccaccaccaccactga

MSDKIIHLTDDSFDTDVLKADGAILVDFWAEWCGPCKMIAPILDEIADEYQGKLTVAKLNIDQNPGTAPKYGIRGIPTLLLFKNGEVAATKVGALSKGQLKEFLDANLAGSGSGHMSDSEVNQEAKPEVKPEVKPETHINLKVSDGSSEIFFKIKKTTPLRRLMEAFAKRQGKEMDSLRFLYDGIRIQADQTPEDLDMEDNDIIEAHREQIGDDDDKAMNNPPPPDDPGAPSPYQRGPDPTVSFVEASRGNYRVATSNVSSLVSGFGGGTIHYPSNATGTMGAIVVIPGYVSGEGSIDWWGPKLASYGFVVMTIGTNSGFDQPPSRARQINNALDYLVEQNTRTGSPVRGMIDPSRLGVIGWSMGGGGTIRVAGEGRIKAAIPLAPWDTSSIPSRGVQAPTLIFACQSDVVAPVRSHASPFYNALPGSISKAFVNLNNGNHFCANGGSSFGRYDAALGRLGVSWMKRFLDEDRRYSQFLCGPNHTGDRQITEYRGNCPYLEHHHHHH*

Candidate 001

atgaatgatcactattcagctctagacacatggaccttggtacgcagctatggtggcggtacgatttactacccgaccgcgaccgaccaaggcacgttcggcgcgattgccatctctccgggtctgaatggtacttggccgggtatcgcgtggttgggtccgcgtctagcgagccaaggtttcgtggtgttcggtatcgagaccaacaacctgaacgacagcccggttagccgcggcacccaactgctggccgcgttggactacctcacccagcgttctagcgttcgcggccgtgtcgatgctggccgcctggctgtggctgggcacagcatgggtggtggaggcgcgctggacgctgcgctgcgtcgtccgtcgctgcaggcagcgatcggcaacgcgccatacctgccgtccggcaacctgtctacggatcgtgttccaaccctggtgtacgcaatgcagaatgataccctggttccgcctagtagattgactacgatgtataacaccatgccggcgaccaccgaaaaagcgtatattgagatcgcaggtgcaggtcataactatatcggtcagccgtccaccactttagcccgtaccatgattccgtggatgaagatctttattgatgacgacgtgcgctacagccagtttctttgcccgctcaccaatcgtgatggtatctcacaatatcgtaatagctgtccgctgattctcgagcaccaccaccaccaccactga

MNDHYSALDTWTLVRSYGGGTIYYPTATDQGTFGAIAISPGLNGTWPGIAWLGPRLASQGFVVFGIETNNLNDSPVSRGTQLLAALDYLTQRSSVRGRVDAGRLAVAGHSMGGGGALDAALRRPSLQAAIGNAPYLPSGNLSTDRVPTLVYAMQNDTLVPPSRLTTMYNTMPATTEKAYIEIAGAGHNYIGQPSTTLARTMIPWMKIFIDDDVRYSQFLCPLTNRDGISQYRNSCPLILEHHHHHH*

Candidate 002

atgatagaaaatggaccctcaccaacactagcggacatccagaaagatggtccgtatgcagtcaccacacagcagattaccggtagcggttttcgtgttggaaccatttatgttccgaccgcggcgggcaaatatgcagttatggcaatctcgccgggtttcggtgcggcgcaatcctctatgcagaagatgggtcagcgtttagctacccatggtttcgtggtcgtgacgatctccacgaacactctgcttgacttcgccgcgagccgcgcatcgcaactcttggctgtgctgaaggcggttacgaccgctcagaccggcccagttgccggtaaggtggatgcgagccgccaggccgtgggcggctggtccatgggcggcggcggtgcgctggaggcagctgctgctgcgccggacctgaaagcgaccgttgcgttcgctccgtgggatttcagcgcaaccaaattcaaaagcattaagatcccgacgtgcatcattggtgcgaccggcgaccagaccgcgcctgtgtcccaatacgctcaaagatactacaacgccatcccgtctacggtgaccaagatgctggcggtcatcaagggcctggaccactttttcccgaccactgcgtctgagccgagcagctataccaatattagttggatgaaacgttttgccgacggcgatatgcgttacagccaatttctgaacgcgcaagatccggcagaagttagcttgaccagcaccggtccgtttctcgagcaccaccaccaccaccactga

MIENGPSPTLADIQKDGPYAVTTQQITGSGFRVGTIYVPTAAGKYAVMAISPGFGAAQSSMQKMGQRLATHGFVVVTISTNTLLDFAASRASQLLAVLKAVTTAQTGPVAGKVDASRQAVGGWSMGGGGALEAAAAAPDLKATVAFAPWDFSATKFKSIKIPTCIIGATGDQTAPVSQYAQRYYNAIPSTVTKMLAVIKGLDHFFPTTASEPSSYTNISWMKRFADGDMRYSQFLNAQDPAEVSLTSTGPFLEHHHHHH*

Candidate 003

atgtcaacaaggaatcaagaacccactctacaatccattgaagcggcgcgcggtccgtatgaaattgcgacccagagcgttccaccggctgtgaataccggcttcggcggaggcaccatttactacccggcgactaccggcgcgggcaaattcggtgccgtcgcattaagcccgggttacatggagaaagaatacagctttagttggctgggtactcgcctggcgtctcagggtttcgtggtgtttatcatcaacacgcaaaacagcgaggaatttccgagcgcacgcactgatgagttgctcgcgtccctgaattatcttacccaaaccagcgaggttcgtgatcgtgtggacccgaaccgtctggctgtgatgggccacagcatgggtggcggcgctgcgttggaggctgcggaaaagaaccagtctttgcgcgcagtgatcagcatgaccccgtggaatatcaagacgacctattctgatattaagaccccgaccctgattatcggcgcgcagtatgataacatcgcccctgttcatgaccacgcccaaccatcgtacgctagcctgccgaacgacctggcgaaaagctacctggagctgcgtaatggtaatcatttttcgccgaacatggaaaataccgcaattgcgcgttccagcaccgtttggctgaaacgttttgtcgatggtgacacccgttataacaagttcatctgtccgggtccgacggttggtgacgagttcaacatcgtacagtccaactgcccgattttgctcgagcaccaccaccaccaccactga

MSTRNQEPTLQSIEAARGPYEIATQSVPPAVNTGFGGGTIYYPATTGAGKFGAVALSPGYMEKEYSFSWLGTRLASQGFVVFIINTQNSEEFPSARTDELLASLNYLTQTSEVRDRVDPNRLAVMGHSMGGGAALEAAEKNQSLRAVISMTPWNIKTTYSDIKTPTLIIGAQYDNIAPVHDHAQPSYASLPNDLAKSYLELRNGNHFSPNMENTAIARSSTVWLKRFVDGDTRYNKFICPGPTVGDEFNIVQSNCPILLEHHHHHH*

Candidate 004

atgtttggcggagggtcaggtgggggaagtacgggtgatgacctgccgcttccggacacttgcacctcgaactgtggttacgaactgggtccgaatccgagcctgagcttcgtgtccgcggcgcaaggtccacagccggtgagcaccgttagcgtttcctcgtctgttgatggttttggtggcggtaccatctactacccgtctaacatctcgcaagagatggcagcgatctccatcgctccgggtttcacgaacacccagagcgcgattgcttggtggggtccgttgctcgcgagtcacggcttcgtcgtgatcaccattgatactaacagcagattcgaccagcctgactcacgtagccgccaactggacagcgcgctgagctatctgattagccaatccgactccagcagcagctcgatctccggcctggtggacgagaaccgcctggccaccatgggcttcagcatgggcggaggcggctcgctgcagtctgcaagccgtaatcgtttgtccgctgccgtcccgctggcaccgtggaattcgggcagcaacagcttcaaccagattgcggtgccgacgatgattatggcctgcgagaatgatagcactgcgaacgttagctcccacgcatctccgttttacaacaccatcccgaccagcaccgataaagcgtttatggagatctctggcggccagcataattgtgcgaatggtagcaactctaacaacgcattgctggctacctatggtgttagttggatgaaacgtttcttggacaaggaccaccgttataaccaatttctgtgcggtccaaatcatgaaagcaacagccgcatttctgaatttcgtgatacctgcgattttaccctcgagcaccaccaccaccaccactga

MFGGGSGGGSTGDDLPLPDTCTSNCGYELGPNPSLSFVSAAQGPQPVSTVSVSSSVDGFGGGTIYYPSNISQEMAAISIAPGFTNTQSAIAWWGPLLASHGFVVITIDTNSRFDQPDSRSRQLDSALSYLISQSDSSSSSISGLVDENRLATMGFSMGGGGSLQSASRNRLSAAVPLAPWNSGSNSFNQIAVPTMIMACENDSTANVSSHASPFYNTIPTSTDKAFMEISGGQHNCANGSNSNNALLATYGVSWMKRFLDKDHRYNQFLCGPNHESNSRISEFRDTCDFTLEHHHHHH*

Candidate 005

atgcaatatcagaaaggacccgatccaacaacgagttctttggagcgcgcaggctcgttcgccacccgtacgattagcgtgtccagcttggcggcgtctggcttcggtggcggccgtatttactacccgaccgcgtccggcagctacggcgtgatcgccgtgagtccgggttacaccggtactgcctctacgatgtacttctggggtgaacgcctgagctcacatggttttgttgttatcgtcatcgaaaccaacacgttactggaccagccgggtagccgtgctaatcagctgatggctgctctcaattataccgtttcccaaggtaacagcagaaccagcgctctttatggtaaggtggatggtaatcgtcgtggtgtcgcaggtcacagcatgggtggcggcggcaccctgattgccgcggctgataacgcgtccctgaaagcggcggttccgatggcgccttggaatctgagctctttgtcgttccgcggtatccgcgtgccgaccatgattttcggctgccaaagcgacgcaatcgcgccagtgaatactcatgcagtcccgttttataacgcgattccgagcagcaccaaaaaaggctacgtggaaatgcgtctggacgaccacttttgtgttatgaacggtggcggccactacgagaccctgggcaagctgggtatcagctggtttaaacgttttatcgatgaagatacccgttattccaagttcctgtgcggtaccgagtacaacgcgaccgttcgtagcaccgaggtttccgacagcaagaacaactgcccgtatctcgagcaccaccaccaccaccactga

MQYQKGPDPTTSSLERAGSFATRTISVSSLAASGFGGGRIYYPTASGSYGVIAVSPGYTGTASTMYFWGERLSSHGFVVIVIETNTLLDQPGSRANQLMAALNYTVSQGNSRTSALYGKVDGNRRGVAGHSMGGGGTLIAAADNASLKAAVPMAPWNLSSLSFRGIRVPTMIFGCQSDAIAPVNTHAVPFYNAIPSSTKKGYVEMRLDDHFCVMNGGGHYETLGKLGISWFKRFIDEDTRYSKFLCGTEYNATVRSTEVSDSKNNCPYLEHHHHHH*

Candidate 006

atgagggatggacccaaatttgggacagctacggtatattacccggagaacgcccaagcgccatacgcaagcctcttgatcgtgactggttttgttcacccggaaagcgcaatgaaagcttggggccctttcctggcgtcccatggtattgtgaccctgaccattggcaccaataaattgaaggacgacccgttcaaaagacgtgatgcgctgctggatgcagttcgtagcctgaaggccgaacacctgcgcaaagactctccgctgtggcataaattggcgctcgaccgcatcggcgttgcgggttggagcatgggtggcggtggcgcacaactggctgctgcagcggatccgtccttacgcgcggtcatcggcttctgcccgtggctgccgaaggctaactttgagcacccggttccgagtttgatcatcgcgggcacgctggacagcattgcgaacgtgttccttaatgcgcgtgcgcactacgctggcatctcggaaaccaccccgaagttgatcatcgagatcccgtatggtggtcattggattgccaacgatccggcgaacgcgaacggtgcaattggtcagattggcctgtcttggctgaaggtgtacctggagggtgatacccgttatcgtcagtttctgttgaccccaccgattagcggtggttattttcgtcacaatctggaactcgagcaccaccaccaccaccactga

MRDGPKFGTATVYYPENAQAPYASLLIVTGFVHPESAMKAWGPFLASHGIVTLTIGTNKLKDDPFKRRDALLDAVRSLKAEHLRKDSPLWHKLALDRIGVAGWSMGGGGAQLAAAADPSLRAVIGFCPWLPKANFEHPVPSLIIAGTLDSIANVFLNARAHYAGISETTPKLIIEIPYGGHWIANDPANANGAIGQIGLSWLKVYLEGDTRYRQFLLTPPISGGYFRHNLELEHHHHHH*

Candidate 007

atggcaagttcagagggagaatctcacgctgagaaaagtgccgaaagcgcaagacacgtggcgccaccgaaaggtcgtcactcttcaagcggtggtagccgccatccgcaccgtcaccgcccagcagagtccggccttgcggcggttagcccgtatcagcgaggcccggatccgacggccgctagcgttgcggcggtcaccggtccgtttgcgatcagctccgttgcagttgctcgcggcaacggcttcggcggcggtgttatttattacccgacggacacctcgcaaggcacctttggtggcatcgccatctccccgggtctgaatggtacgtggccgggtattgcttggctgggcccgcgtttggcgtcgcaaggtttcgtggtgtttggcatcgagacaaataacctcaacgacagcccggcgtctagaggtacccagctgctggccgctctcgactacctgactcgcagctccaccgttcgtacccgtgtggacgcgggtcgtctgggtgtgattggtcacagcatgggtggtgggggcgcgctggatgccgcgctgcgtcgtccggcgctgcaggcggcgatcggcaacgcaccgcatctgccgagcggtagcctggctggcgatcgcgtgccgactttggtttacgcaatgcaaaatgataccttggttccgcctacgcgtttgacctcattatacaataccattccagctgcgaccgaacgtgcatatattgaagtggcgggtgccggtcataactacatcggccagccgtccaccgtactggctcgtaccatgattccgtggttgaagatcttcatcgatgacgacgctcgctacagccagttcctgtgcccgctgagcaaccaggcaggcatcacccaatatcgtagctcttgtccgctgatttcgaccactgccaccgtctctcgtggaccgctcgagcaccaccaccaccaccactga

MASSEGESHAEKSAESARHVAPPKGRHSSSGGSRHPHRHRPAESGLAAVSPYQRGPDPTAASVAAVTGPFAISSVAVARGNGFGGGVIYYPTDTSQGTFGGIAISPGLNGTWPGIAWLGPRLASQGFVVFGIETNNLNDSPASRGTQLLAALDYLTRSSTVRTRVDAGRLGVIGHSMGGGGALDAALRRPALQAAIGNAPHLPSGSLAGDRVPTLVYAMQNDTLVPPTRLTSLYNTIPAATERAYIEVAGAGHNYIGQPSTVLARTMIPWLKIFIDDDARYSQFLCPLSNQAGITQYRSSCPLISTTATVSRGPLEHHHHHH*

Candidate 008

atgtcaggatttgggggaggtacaatatatttcccgagcaatgcccaacaggagctggcggcgattgccgtcgcgccaggttttaccaactaccagagcgcgattcagtggtggggtcctctgttggcgagtcacggctatgttgtgatcaccatcgacaccaatggtcgtttcgataatccgaattcccgctctctccagctggatgcggcactgagccatctgatcgacgagggtaatcgcgcaaactcgccgatttccggcatcgtagatgagaaccgcctggcgaccatgggtttcagcatgggcggtggcggtagcttaatcagcgctagccgtaatcgtttgtctgcagctgtgccgctggcgccgtggaatagcggcggaaacgacttcgaccaaattggtgttccgacgatgatcatggcgtgcgaaaacgataccactgccagcgttaattcacacgcatcgccgttttataacaccattccgaactctaccgataaggcgtacatggaaattaccaacggtccgcataactgcgcgaatggcgataacggcaacggtaacgacgccttgctgtccacgtacggcgtgagctggatgaaaagatttctggacaaggaccagcgttatgctcaatttctttgcggtccgaaccacatcgctaacccgtccatcaacgaataccgtgataactgtaattacctcgagcaccaccaccaccaccactga

MSGFGGGTIYFPSNAQQELAAIAVAPGFTNYQSAIQWWGPLLASHGYVVITIDTNGRFDNPNSRSLQLDAALSHLIDEGNRANSPISGIVDENRLATMGFSMGGGGSLISASRNRLSAAVPLAPWNSGGNDFDQIGVPTMIMACENDTTASVNSHASPFYNTIPNSTDKAYMEITNGPHNCANGDNGNGNDALLSTYGVSWMKRFLDKDQRYAQFLCGPNHIANPSINEYRDNCNYLEHHHHHH*

Candidate 009

atggtacaaaacggaccaaatcccacactagacgcaatcaaagcagacggtccgtacgcaattacctcgcaagcggtggctggcaacggctttggtgctggtacaatttatatgccgaccgcagccggcaaatacgccctggttgcgatctgcccgggcttcgtgagcgcgcaaagcaccatggcgggcatggcgaaacgcctggcgacccatggtttcgttgtggtggccatcagcacgaacaccctgtttgattttccgagctcccgcgcaacgcagctgctcgctgcgcttaagaccgttagcgcattgaacaccggtgccgtggttggtaagatcgacaccacgcgtcaggctgtgagcggttggtcaatgggtggcggtggcaccttggaggctgcgggcgctactccgggtctggtagcggcggtcgcgtttgctccgtgggatacgtccaccaccaagatgaaggtgaataaggttccggcagccattatcggcggtagctctgatgttatcgcccctgttgcgtcccacagccagaaattctatgatgcgattccggcgacgactccgaaactgctggcggtcatcaacggcgccgaccacttcttcccaaccaaggcgtctgaaccggcttcctataccaatattagctggatgaaacgttttgcggacggcgatacccgttacagcagttttctgaatggaaacgacgcggcgttgtcttcgttcagctccaacggtccgttcctcgagcaccaccaccaccaccactga

MVQNGPNPTLDAIKADGPYAITSQAVAGNGFGAGTIYMPTAAGKYALVAICPGFVSAQSTMAGMAKRLATHGFVVVAISTNTLFDFPSSRATQLLAALKTVSALNTGAVVGKIDTTRQAVSGWSMGGGGTLEAAGATPGLVAAVAFAPWDTSTTKMKVNKVPAAIIGGSSDVIAPVASHSQKFYDAIPATTPKLLAVINGADHFFPTKASEPASYTNISWMKRFADGDTRYSSFLNGNDAALSSFSSNGPFLEHHHHHH*

Candidate 010

atgcagacaaatccatatcaaaaaggacccgcaccaaccgtcgcgaacttggaggcagcgcgtggttcgttcgccgttagctctacgtcgatcagctccttgtctgcaaccggctttggtggcggtagccttcactacccgactgcggcgggaacctacggcctgatcgctgtggcgccgccttatctcgctcaaagctcctctatcgcgtggctgggtccgcgtctggctagccatggtttcgtggtggttatgattgacgtaaattccaccctggattttccggaaagccgtagccgtcagcagctggctgccatcgattatgcgatcgcgcagaacgcgcgccgcacctctccgatttatggcaaggtggacaccacccgtcgcggcgttaccggtcatagcatgggtggcggggcgagcatcctgaccgcgcgcaacagcccggccaacattaaaggcgcgtttccgctgaccccttggtcaagcgacaagaacttcagcaatttgcaagtgccgacgggctacttagcctgccaagatgataccgttgcgggtaatgcggaccactccttgaatttctataaccgtatgccggcatccaccccgaaaggttactacgagctaagaggcgaaacccacttttgtccgcagagcacgacgaacaacccggttattggtaagtacctggttgcatggatgaaacgtttcgtcgacaacgatactcgcttctccccatttctgagcggtgcgctgccgaatgccgacctgtcttccggccgtttctcggtgctgcgtaccagcggtctgctcgagcaccaccaccaccaccactga

MQTNPYQKGPAPTVANLEAARGSFAVSSTSISSLSATGFGGGSLHYPTAAGTYGLIAVAPPYLAQSSSIAWLGPRLASHGFVVVMIDVNSTLDFPESRSRQQLAAIDYAIAQNARRTSPIYGKVDTTRRGVTGHSMGGGASILTARNSPANIKGAFPLTPWSSDKNFSNLQVPTGYLACQDDTVAGNADHSLNFYNRMPASTPKGYYELRGETHFCPQSTTNNPVIGKYLVAWMKRFVDNDTRFSPFLSGALPNADLSSGRFSVLRTSGLLEHHHHHH*

Candidate 011 (AhPETase)

atggcttcaccctatgaaaggggaccaaatccgacggctgcgtcagtaagcgcaaccacaggtccgtttgcaattgcctctgtgtctgtcgccagaggcaacggtttcggcggcggcgttatttactacccgacggcgacgaaccagggcaccttcggcggtattgccatcagcccgggtttgaatggtacttgggcgggcatcgcgtggctgggcccgcgtctggcgtcgcaaggctttgttgtctttggcattgaaaccaacaacctgaacgatagcccgaccagtcgcggtacgcaactgctggctgcgctggactacctgacccagcgttcgaccgttcgtacccgcgtggatgctgggcgtttggctgtgatgggtcacagcatgggcggaggtggcgcgttggacgcggctctgcgtcgccctagcctgcaggcggcgatcggtaatgcaccacatctgccgtccggtacgctctccaacgaccgtgtgccgacgatgatctacgcaatgcagaacgatactcttgttccaccgtcccgtttgaccaccctgtataacagcattccggcaaccaccgagcgcggttatatcctggtgaccggtgcgggccacaattatattggtcagccgtccaccaccttagcgcgtaccatgattccgtggttcaagatcttcatcgatgacgacacccgctacagccagtttttgtgcccgatgagcaatcaaagcggtatcagccaatatcgtaattcttgtccgctgagcctcgagcaccaccaccaccaccactga

MASPYERGPNPTAASVSATTGPFAIASVSVARGNGFGGGVIYYPTATNQGTFGGIAISPGLNGTWAGIAWLGPRLASQGFVVFGIETNNLNDSPTSRGTQLLAALDYLTQRSTVRTRVDAGRLAVMGHSMGGGGALDAALRRPSLQAAIGNAPHLPSGTLSNDRVPTMIYAMQNDTLVPPSRLTTLYNSIPATTERGYILVTGAGHNYIGQPSTTLARTMIPWFKIFIDDDTRYSQFLCPMSNQSGISQYRNSCPLSLEHHHHHH*

Candidate 012

atgcaagtacacggacccgatccaacagttaagagcttggaagcggaggcaggtccgtacaccgtcgcatcggcgaaagttggttctccgagcggttatggtgccggtacggtgtattacccgaccgtgcgtaccgagggccagtttggcgtggtggtgctggcccctggtttcctggcgattcaaaactattacaaatggctggcggagcgtgttgcgtcccatggtttcgtggtggtcaccgttaatttcaactccgtgtttgatcagccggaaagcagaggcacgcagatgggcaaagcattgcaacaggttgtaagcctgagcaagaccatgaccacgccatactacggcgttttggatgctaatcgtcaggcagctatgggccactctatgggtggcggcggcaccctggcggctgcgcgcaacaccccgagtcttaaggcggctgtggctctggcgccgtggcataccacaaaggactggagcgcattacaagttccgacgttggttctggctgacgaaaaagacagcatcgcgaccaatgatcaacactccatcccgttctatcaaagctttaacgcagcgctgccgtcggcgtatatcgagctggctggcgcggaccacctgtgtggtattcagatcagcgccaccaagtgcaaaactgccctcgcgaaatacagcattgcatggctgaagcgctttgttgatggtgacgcgcgttataccccgttcgtcaaaactcgcgcagccgagatgtccgcgtttgaaacccgtggtgcgtacctcgagcaccaccaccaccaccactga

MQVHGPDPTVKSLEAEAGPYTVASAKVGSPSGYGAGTVYYPTVRTEGQFGVVVLAPGFLAIQNYYKWLAERVASHGFVVVTVNFNSVFDQPESRGTQMGKALQQVVSLSKTMTTPYYGVLDANRQAAMGHSMGGGGTLAAARNTPSLKAAVALAPWHTTKDWSALQVPTLVLADEKDSIATNDQHSIPFYQSFNAALPSAYIELAGADHLCGIQISATKCKTALAKYSIAWLKRFVDGDARYTPFVKTRAAEMSAFETRGAYLEHHHHHH*

Candidate 013

atggatccagctccccacggacccgaccctacggcctccttgcttgaggcaaagcaaggtccatttgcagtggcaaataccaaagttgctagcccgtccggctacggcgcggccaccgtaacctacccgactgcgaaaagcgatggtccgtatggtctggttgtcttggcgccaggttttctgctggcgcagggctattatagctggctggccgaacgtgttgctagccacggcttcgtggttgtgaacctgtccaccaatagcatttttgacagcccggacgtgagagcaccgcagttagcgagcgcgcttaagcaagttctggcgttgtcgcaaaccgcgaccagcccgtactctggtctggttaacccggatcgcctcgcgctgatgggtcatagcgccggcggtggtgcggctttgaccgcggcgttgtcgaacccgggtctgaaggctgtcgtcggtctcaccctggcggttggcacccaaaaagagttcagccagctgcaggtgccggcactggtgttggctgcggagaaggacgttattgcgcccaacgcgacgtactctatgccggtttttcagagcctgtcggctgcgctgccgagcgcctatatcgaaattgcaggcgcagatcatttgaccccgacgatcctggagggttctcgtatgcgtgccgtgttaggccgttataccatcgcatggctgaagcgcttcgtggatgaagaccgccgttacactccgttcacccagggcagccgtccggaactgtccaaatacctgtctcacggggcgtggctcgagcaccaccaccaccaccactga

MDPAPHGPDPTASLLEAKQGPFAVANTKVASPSGYGAATVTYPTAKSDGPYGLVVLAPGFLLAQGYYSWLAERVASHGFVVVNLSTNSIFDSPDVRAPQLASALKQVLALSQTATSPYSGLVNPDRLALMGHSAGGGAALTAALSNPGLKAVVGLTLAVGTQKEFSQLQVPALVLAAEKDVIAPNATYSMPVFQSLSAALPSAYIEIAGADHLTPTILEGSRMRAVLGRYTIAWLKRFVDEDRRYTPFTQGSRPELSKYLSHGAWLEHHHHHH*

Candidate 014

atggtaacaaaaggaccagatccctcagctgcggaagtggcaaaggtgactggtagctatgctgtgacggaaaccaaagttgatgacgccgcgtcgacgaaattcggcccagcgaccgtgtatgcaccgaaaggtgcgcctggtgaaacctttggtgccatcgcgatcagcccgggtttcctggcagagcagtggaccttaacctggttggctcaacgtctgtcctcttttggctttgttaccattgtttttacccccaacagcatttttgatcagccagacgcgcgtagccgtgcgttgctggacgccctggagtacctgaaaacccagtctacggcaaaggcgctgattgatccgagccgcctgggcgtactgggtcacagcatgggtggcggcggcgcattggaggcagctagtagagatctgacccttaaggctgctgtggcgttgctcccgtgggatctgactaagaacttcagcggtgttcgtaccccgaccttggtcatcggcgcgaaaccggatttcatcgctccggtggcgtcccatgcgaagccgttctacgcgtcgctgagcacctccctgccgcgcgcgtacttggagctgaatattggccacgctcaaccggcactggttccgacgaccgaagttagccgcgcagcggtcaactggttcaaacgttttctggacgacgacccgcgttataccaagaccttatgcccggcggtggccggtgttaatgcatccaccgttacggcgtaccagagcagctgtggtcaaggtccgctgtctctcgagcaccaccaccaccaccactga

MVTKGPDPSAAEVAKVTGSYAVTETKVDDAASTKFGPATVYAPKGAPGETFGAIAISPGFLAEQWTLTWLAQRLSSFGFVTIVFTPNSIFDQPDARSRALLDALEYLKTQSTAKALIDPSRLGVLGHSMGGGGALEAASRDLTLKAAVALLPWDLTKNFSGVRTPTLVIGAKPDFIAPVASHAKPFYASLSTSLPRAYLELNIGHAQPALVPTTEVSRAAVNWFKRFLDDDPRYTKTLCPAVAGVNASTVTAYQSSCGQGPLSLEHHHHHH*

Candidate 015

atgcccgtaaggtcaagtgcttctgttcaatcgaaaccgtctctctcccgttacagcaccatccgtgcgacgtaccgcgctggtcagtggatgggtatgcgtgccgactgggatctgtaccaaggccgtgcgggtctgcgtggtaatgataactggccgctgcgtaatgagaagagaacgagctcccgcattcagcgtatgaacagccatcagttcgatggtttgtctacgcgtggcttcaaaatgtttaccaaacaacacaaattcagccgccaagtatccttggctctggcagctgcggctttggtagcgaccttcagcagtccggttcacgcgggcagcgcattcgaacgtggcccggacccgactgtcgagagcgtgtcacaaaaaggtccgtttgacatcgatgcgtacgtgatcgcccgtgcgaacgccaaaggctatggtggcgcgacggtttattacccgaaaagcggtacacagacctttggtgttgtgtcgatcaccccaggtttcctggcgtcccaagcgaactataaaccgttggctcagtttgttgcaagccatggtttcgtggttatcaatctcgacccggttaccatttttgaccagccggatgcacgagcgaacgaaatggcagcggcgttgaagcaggtggtgggcatggttaacaccggtaaggtgccgttcgccgtcgtgaccgatgtttctagacgtgctgtgtctggccacagcatgggcggtggaggcacccttatggccgcgactgcggacccttccctgaaggcagcggttccgctggctccgtggagcttggtcaagagctttgcgcgcgatgcggttccgacgatgattgtcgcatgccagaaggataccattgcgaccccgtcccaacacgccgacctgttttatgccagcctgccagcaaacctgccgcgcggtgaagtcgaggttaagggcgtggaccacatgtgtagcaccaattacgcaccggcgtatgtgaacgaggttggtatggttgtggttagttggctgaagcgtttcctggacgaagatatgcgttacgacagcttagttaaggacggcctgaataccaccggcgattttagccgcttcgacgtgcagggtttcctcgagcaccaccaccaccaccactga

MPVRSSASVQSKPSLSRYSTIRATYRAGQWMGMRADWDLYQGRAGLRGNDNWPLRNEKRTSSRIQRMNSHQFDGLSTRGFKMFTKQHKFSRQVSLALAAAALVATFSSPVHAGSAFERGPDPTVESVSQKGPFDIDAYVIARANAKGYGGATVYYPKSGTQTFGVVSITPGFLASQANYKPLAQFVASHGFVVINLDPVTIFDQPDARANEMAAALKQVVGMVNTGKVPFAVVTDVSRRAVSGHSMGGGGTLMAATADPSLKAAVPLAPWSLVKSFARDAVPTMIVACQKDTIATPSQHADLFYASLPANLPRGEVEVKGVDHMCSTNYAPAYVNEVGMVVVSWLKRFLDEDMRYDSLVKDGLNTTGDFSRFDVQGFLEHHHHHH*

Candidate 016

atggatgtaaatccatttcaaaggggacccgacccgactgccgctggcgttgccgctgcccgcggcccgttcgccaccgcacaggttaccgtaccagcaggtaacggcttcggcggtggtgtcgtgtattacccgactgacacctcgcaaggtacatttggtgccatcgccatcgcgccaggttacggcaccgcatggagctactacgcgtggcagggccctagactggcgagctttggcttcgtggttattggtattgaaaccaatacgctgaacgactacgcggatgctcgcgcagcgcaactgttggcggcgttggactggctggttacctccagcccggtccgtgatcgtgtcgatcgtaatcgtctggctgtgggtggccacagcatgggtggtggcggcgctttgctcgcggcgatccagcgtccgagcctgatgaccgcgatcggcatggcaccgtatgttccgggtggtgatttgtctggtatccgtattccgaccgtgctgttcgcgggtcagaccgacggtacggtgaccccgagctatgcgctgtccgcgtataacaccattccgggtagcgttgagcgcgcatacgtggagatcgcgaatgaaggtcatggttttccggcaggcggcggcggcggaaacagcggtgcgtttgcacgcaccgagatgatttggatgaagctgtttattgataaagatacccgttacgctccgttcctgtgtccgagtcttagcaacatgaacggcatcagccgttatcaagcgagctgcccgctggacccgcctggtggcaccaccccaacgccgtctgctaccccgacgtccagcccgacgtcaaccccgacgtccaccccgtcggcgacctctgccttactcgagcaccaccaccaccaccactga

MDVNPFQRGPDPTAAGVAAARGPFATAQVTVPAGNGFGGGVVYYPTDTSQGTFGAIAIAPGYGTAWSYYAWQGPRLASFGFVVIGIETNTLNDYADARAAQLLAALDWLVTSSPVRDRVDRNRLAVGGHSMGGGGALLAAIQRPSLMTAIGMAPYVPGGDLSGIRIPTVLFAGQTDGTVTPSYALSAYNTIPGSVERAYVEIANEGHGFPAGGGGGNSGAFARTEMIWMKLFIDKDTRYAPFLCPSLSNMNGISRYQASCPLDPPGGTTPTPSATPTSSPTSTPTSTPSATSALLEHHHHHH*

Candidate 017

atggaaccctcagctagtgtaaaagcaacacaacgtggtccggacccgacgttagctatgatcgaggcgagccgcggtccgttcgccaccgcgcagcaaaacgttagcccgggaaacggcttcaatggcggcgtggtgtactacccgacggataccagcctgggcacctggggtgcattggcgattgttccgggttataccgctaagtgcgcgaaagaagaggcttggatgggtccttggctgtcgtcttttggtttcgtggtgatttgtattgagacaaatagcccgaatgattgggatacggcgcgtggtcagcaactgctggccgcgcttgattggttgaccaccagaagcccggtcaaaaaccgtgttgaccccaaccgcctgtctgttctgggtcattccatgggtggcggcgggatggtgtacgcgaccgagcaccgccctaccctgaaggccggtattggcctggcaccgttcagcccatctcagaatatgagcagcgaacgtgtcccaaccctggttttgggcggccagaaagacccgactgtaactccgtcctatctgaagggtctgtacgcgggtgttccggcatcgaccaaatccttgtttgctgaaatcagcggtgcggaccacctgtactacacccatccgaacaacgtgcagatgaaaatcatcattccgtggctcaagatctttgttgaccaggatagccgttatcaacaatttctttgcccgaaactgccggacccgaccaagatctccatgtatcaggcaaagtgcccgtatatcctcgagcaccaccaccaccaccactga

MEPSASVKATQRGPDPTLAMIEASRGPFATAQQNVSPGNGFNGGVVYYPTDTSLGTWGALAIVPGYTAKCAKEEAWMGPWLSSFGFVVICIETNSPNDWDTARGQQLLAALDWLTTRSPVKNRVDPNRLSVLGHSMGGGGMVYATEHRPTLKAGIGLAPFSPSQNMSSERVPTLVLGGQKDPTVTPSYLKGLYAGVPASTKSLFAEISGADHLYYTHPNNVQMKIIIPWLKIFVDQDSRYQQFLCPKLPDPTKISMYQAKCPYILEHHHHHH*

Candidate 018

atgggatttaatgggggtacaatatactatccgaccgacacctccatgggcacctggggtgcggttgcgatcgtgccgggttatacggcgctgtttgcaaatgaggaagcgtggatgggtccgtggctgagcagcttcggtttcgttgtcatcggtattgaaaccaacagccgtaccgactacgacactgcgagaggtacggagttgctggctgccctggactacctgacccagaaaagcccggtaaaagatcgcgttgatccgtctcgtctgtccgttatcggccacagcatgggcggtggcggcgtggtctatgccaccgagcaccgcccaagcctgaaggctgctgtggcattggcccctttttcgccgtcccaaaatatgagcaccgacaccgtgccaacgatggtgatgggcggccagaacgacactgttgttaccccgtcatatttggatggtctctacgcgaccatgccggcatctacccaaagcgatttcgtgcagatcgcgggtgcggatcatgtttggtacacccatccggaaaccctggagatgcgtattctgattccgtggttaaagaccttcgtggacaacgatacgcgttatacgcaatttctttgcccgctcgagcaccaccaccaccaccactga

MGFNGGTIYYPTDTSMGTWGAVAIVPGYTALFANEEAWMGPWLSSFGFVVIGIETNSRTDYDTARGTELLAALDYLTQKSPVKDRVDPSRLSVIGHSMGGGGVVYATEHRPSLKAAVALAPFSPSQNMSTDTVPTMVMGGQNDTVVTPSYLDGLYATMPASTQSDFVQIAGADHVWYTHPETLEMRILIPWLKTFVDNDTRYTQFLCPLEHHHHHH*

Candidate 019

atgtttaatcccgggtcaggtggaggaagtggtggcgcgaacacgtgcgaaaccaattgtggcttcgagcgcggtccggatccgaccttgtctggcttgcgttccggcaccggcccatacagcgtgagcaccgtggacgtgccgtatggtagcgcgggttttggtgggggcaccattcattatccgaccaacacttcaggcggcaaaatgggcgcgatcgctgtgattccgggtttcgtctctccggagtcgagcattggttggtggggtccggcactggcgagtcaaggattcgtggttctgaccatgggtacccagaccccgttggacagcccgagcgctcgcgctcgccagttaaccgcaggcctggaccaccttgttgaacaaagcaacagcagctcttcggcgattagcggcctcgtggacccgagccgtgtcgctatggttggttggagcatgggtggtggcggcacgctgaagaatgcggccggtgatcgtctgagcgctgcgatcccgctggcaccgtttgacatggatatgagctcttacaagaacatcgaaactccgaccatgatcctggggtgcgagtccgatatcatcgccgcggcggatctgttcgccgtacctagctacgagcacattccggagtccaccgataaggcatatttcgaattcgcgggtcagggtcattggtgcgcgaatggtaataacgacaacaacgaactgctgagcatcttgggcgtttcctggatgaaaagatatctggacaaagacacgcgttacgaccagtttctgtgtggtacgaaccactcctcgaactcggcgttttcccaagttcgtgatacctgcaatctcgagcaccaccaccaccaccactga

MFNPGSGGGSGGANTCETNCGFERGPDPTLSGLRSGTGPYSVSTVDVPYGSAGFGGGTIHYPTNTSGGKMGAIAVIPGFVSPESSIGWWGPALASQGFVVLTMGTQTPLDSPSARARQLTAGLDHLVEQSNSSSSAISGLVDPSRVAMVGWSMGGGGTLKNAAGDRLSAAIPLAPFDMDMSSYKNIETPTMILGCESDIIAAADLFAVPSYEHIPESTDKAYFEFAGQGHWCANGNNDNNELLSILGVSWMKRYLDKDTRYDQFLCGTNHSSNSAFSQVRDTCNLEHHHHHH*

Candidate 020

atgtcaaattttacacccttcgcttttgattcgatcgtggagagcaatggcctgcgtaatggtccgaattacgacggcgccaccctcttcttcccagtgaatgagaacaccaactacaaaagcattgtgttggtgccgggttataaggcggaacaagagtcggtcaaacgttgggcgcgttatctggcgagccgcggttttatcagcatgaccatcggcaccaactcgctgaccgattttccgtctgttcgtgctaatgctctgatcgacggcatggaaaccattagacaagagaactctcgccaaagctctccgctgtatcagaaaatcgacaccaacaacattgcagttggtggctggagtatgggtggcggtggcgcgcagctggccgcaaaaatggatccacgtattaaagcggttttggcgattaccccgtggctgtacaagtacaccctgagcgcgtccgacctgaaccacaactccccggtgcttatcatcagcggtgaagttgatgcgacggctccggtcagcaagcatgctgatatccactataactacactccgtcctctacgaacaaaatgctgttcgaggttttaggtggcggccataacaagccgttgaagccgttgcgccgtaaaggtgttctgggcaatgttgcgtacgcgtggttgaagctgttcttggaaggtaataactgctactgcggtattctgtccgtggacagctttgatcagaattccaccagcagcaagtacctgacgaacctgaattgtagcagcttgctcgagcaccaccaccaccaccactga

MSNFTPFAFDSIVESNGLRNGPNYDGATLFFPVNENTNYKSIVLVPGYKAEQESVKRWARYLASRGFISMTIGTNSLTDFPSVRANALIDGMETIRQENSRQSSPLYQKIDTNNIAVGGWSMGGGGAQLAAKMDPRIKAVLAITPWLYKYTLSASDLNHNSPVLIISGEVDATAPVSKHADIHYNYTPSSTNKMLFEVLGGGHNKPLKPLRRKGVLGNVAYAWLKLFLEGNNCYCGILSVDSFDQNSTSSKYLTNLNCSSLLEHHHHHH*

**Table S5 Primers used in library construction**

| **Name** | **Sequence** |
| --- | --- |
| H154NNK-TOP | ATCGGTAATGCACCANNKCTGCCGTCCGG |
| H154NNK-BOT | TGGTGCATTACCGATCGCCGCCTGC |
| D163NNK-TOP | GGTACGCTCTCCAACNNKCGTGTGCCGA |
| D163NNK-BOT | GTTGGAGAGCGTACCGGACGGCAGAT |
| Y170NNK-TOP | GTGCCGACGATGATCNNKGCAATGCAGAAC |
| Y170NNK-BOT | GATCATCGTCGGCACACGGTCGTTGGA |
| M172NNK-TOP | ACGATGATCTACGCANNKCAGAACGATACT |
| M172NNK-BOT | TGCGTAGATCATCGTCGGCACACG |
| L90T-TOP | CATTGAAACCAACAACACGAACGATAGCCCGACC |
| L90T-BOT | GTTGTTGGTTTCAATGCCAAAGACAACA |
| P156T-TOP | TAATGCACCACATCTGACGTCCGGTACGC |
| P156T-BOT | CAGATGTGGTGCATTACCGATCGCCGC |
| Plas-S248C-TOP | CAATATCGTAATTCTTGTCCGCTGAGCCTCGAGCA |
| Plas-T202C-BOT | CACCAGGATATAACCGCGCTCGGT |
| S248C | AAGAATTACGATATTGGCAGATACCGCT |
| T202C | GGTTATATCCTGGTGTGCGGTGCGGGCCAC |
| H154F-TOP | ATCGGTAATGCACCATTCCTGCCGTCCGG |
| H154W-TOP | ATCGGTAATGCACCATGGCTGCCGTCCGG |
| G210N-TOP | GGCCACAATTATATTAACCAGCCGTCCACCA |
| G210N-BOT | AATATAATTGTGGCCCGCACCGGT |
| S93Q-TOP | AACAACCTGAACGATCAACCGACCAGTCGC |
| S93Q-BOT | ATCGTTCAGGTTGTTGGTTTCAATGCCAAAGACAAC |
| L61F-TOP | GCCATCAGCCCGGGTTTCAATGGTACTTGG |
| L61Y-TOP | GCCATCAGCCCGGGTTACAATGGTACTTGG |
| L61-BOT | ACCCGGGCTGATGGCAATACCGC |
| N207F-TOP | ACCGGTGCGGGCCACTTCTATATTGGTCAG |
| N207F-BOT | GTGGCCCGCACCGGTCACCA |
| S93Y-TOP | AACAACCTGAACGATTACCCGACCAGTCGC |
| S93Y-BOT | TCGTTCAGGTTGTTGGTTTCAATGCCAAAGACAA |
| F154Y-TOP | ATCGGTAATGCACCATACCTGCCGTCCGGT |
| F154Y-BOT | TGGTGCATTACCGATCGCCGCCT |
| L61A-TOP | GCCATCAGCCCGGGTGCAAATGGTACTTGG |
| L61V-TOP | GCCATCAGCCCGGGTGTAAATGGTACTTGG |
| S93G-TOP | AACAACCTGAACGATGGACCGACCAGTCGC |
| S93G-BOT | ATCGTTCAGGTTGTTGGTTTCAATGCCAAA |
| S93NNK-TOP | AACAACCTGAACGATNNKCCGACCAGTCGC |
| S93NNK-BOT | ATCGTTCAAGGTTGTTGGTTTCAATGGCCAAA |
| G210NNK-TOP | GGCCACAATTATATTNNKCAGCCGTCCACC |
| G21ONNK-BOT | AATATAATTGTGGCCCGCACCGGTCA |
| pET32a-SUMO-BOT | ACTTCTGAGTCCGACATATGGCCAGAACCAGAAC |
| pET32a-TOP | ACTGAGATCCGGCTGCTAACAAAGCCCG |
| MG8-pET32a | TTAGCAGCCGGATCTCAGTGGTGGTGGTGGTGGT |
| SUMO-MG8 | GTGACGACGACGACAAGGCCATGAACAATCCGC |
| SUMO-BOT | CTTGTCGTCGTCGTCACCAATCTGTTCTCTGTGAGC |
| SUMO-TOP | TGGTTCTGGCCATATGTCGGACTCAGAAGTCAAT |
| pET32a-BOT | GTCCGACATATGGCCAGAACCAGAACCGGC |

**Table S6 Summary of Bioinformatics and Computational Tools**

| Tool type | Tool name | Version | Reference |
| --- | --- | --- | --- |
| Phylogenetic & Sequence Analysis | SnapGene | 8.2.2 | www.snapgene.com |
|  | MEGA | 12.0.11 | 10.1093/molbev/msae263 |
|  | iTOL | v6 | 10.1093/nar/gkae268 |
|  | ESPrit | 3.0 | 10.1093/nar/gkg556 |
|  | SignalP | 6.0 | 10.1038/s41587-021-01156-3 |
| Structural Biology & Molecular Dynamics | PyMOL | 3.1.4.1 | / |
|  | Boltz | 2.0 | 10.1101/2025.06.14.659707 |
|  | GROMACS | 2022.2-plumed_2.8.3 | 10.1016/j.softx.2015.06.001 |
| Machine Learning & Zero-Shot Predictors | EVE | / | 10.1038/s41586-021-04043-8 |
|  | EVmutation | v2.evcouplings.org | 10.1093/bioinformatics/bty862 |
|  | MSA Transformer | esm_msa1b_t12_100M_UR50S () | 10.1101/2021.02.12.430858 |
|  | Tranception | / | 10.48550/arXiv.2205.13760 |
|  | ESM-1v | esm1v_t33_650M_UR90S_1-5 () | 10.1101/2021.07.09.450648 |
|  | ESM-2 | esm2_t36_150M-15B_UR50D () | 10.1126/science.ade2574 |
|  | SaProt | SaProt_650M_PDB | 10.1101/2023.10.01.560349 |

**Table S7 Quantification of Pc-PET MPs degradation products under actual 48 h cell exposure conditions**

Under cell-culture-mimicking conditions (MEM media, 37 °C), AhPETase^M1^ generated approximately 2.32 ± 0.06 mM main PET depolymerization products (TPA: 1.78 mM, MHET: 0.54 mM) within 48 h, confirming that the enzyme actively depolymerizes Pc-PET MPs during the same time frame used for the cytotoxicity assays and well below the concentration at which the product becomes cytotoxic.

**Table S8 Crystallinity of four raw PET materials and Pc-PET MPs**

**Supplementary Note 1: Criteria for establishing the concentration gradient of PET MPs to assess Pc-PET MP toxicity and the potential detoxifying effect of AhPETase^M1^.**

The concentration gradient of 10 to 1,000 μg/mL was carefully established based on a combination of recent toxicological consensus, physiological accumulation scenarios, and the specific requirement to evaluate enzymatic rescue efficacy. The detailed criteria are as follows:

1. The lower bound (10 μg/mL) as the threshold of toxicity

While baseline environmental and blood concentrations of MPs are typically in the lower μg/mL range, chronic exposure leads to significant tissue accumulation (e.g., in the gut and lungs). According to a comprehensive meta-analysis of MP toxicological impacts on human cells by Danopoulos et al. [1], 10 μg/mL represents the widely accepted lowest observed adverse effect level. At this threshold, MPs begin to disrupt cell barriers physically and chemically and reduce cell viability. A recent study by Manoochehri et al. [2] specifically investigating PET nanoplastics also confirmed that cytotoxicity and oxidative stress in human cells initiate at 10 μg/mL. Therefore, we selected 10 μg/mL as our starting concentration to reflect a realistic high-exposure physiological accumulation scenario.

2. The upper bound (1,000 μg/mL) for robust toxicological profiling

The aforementioned study [2] demonstrated that the IC50 for PET plastics in HepG2 cell lines is approximately 616.7 μg/mL. To ensure we captured the full spectrum of cytotoxicity, from mild inhibition to severe acute toxicity, we extended our upper gradient to 1,000 μg/mL.

3. Establishing a baseline for enzymatic detoxification

Crucially, the primary objective of our cell experiments was to demonstrate the proof-of-concept for enzymatic detoxification. To unequivocally prove that AhPETase^M1^ can "rescue" cells, we first needed to induce a robust, statistically significant, and reproducible cell damage model. The 1,000 μg/mL concentration successfully reduced cell viability to ~70-74%, providing an ideal and stringent baseline to measure the restorative efficacy of our engineered enzyme.

References for this note:

[1] Danopoulos, E. et al. (2022) A rapid review and meta-regression analyses of the toxicological impacts of microplastic exposure in human cells. ***J. Hazard. Mater.*** 427. 10.1016/j.jhazmat.2021.127861

[2] Manoochehri, Z. et al. (2024) In vitro toxicity of polyethylene terephthalate nanoplastics (PET-NPs) in human hepatocarcinoma (HepG2) cell line. ***Toxicol. Environ. Health Sci.*** 16, 203-215. 10.1007/s13530-024-00213-z

**Supplementary Note 2: An in-depth explanation of the physicochemical alterations to PET following TFA treatment, specifically focusing on changes in crystallinity and ester bond integrity.**

1. Alterations to crystallinity (physical change)

The TFA dissolution/precipitation process significantly alters the crystallinity of the PET fragments. The initial post-consumer PET fragments possess a highly ordered crystalline structure. TFA acts as a strong solvent that disrupts the intermolecular hydrogen bonding and van der Waals forces between polymer chains.

Upon rapid precipitation with water, the polymer chains do not have sufficient time to re-align into ordered crystalline lattices, resulting in precipitated MPs that are predominantly amorphous with significantly reduced crystallinity. However, this physical transition makes the substrate more susceptible to enzymatic attack, and it is a necessary step to generate uniform microplastics for downstream screening and cellular assays.

2. Integrity of main-chain ester bonds (no depolymerization)

Regarding the chemical backbone, the TFA treatment does not induce the breaking of main-chain ester bonds (i.e., no macroscopic depolymerization). According to comprehensive reviews on PET chemical recycling [1], the cleavage of PET's stable aromatic ester backbone requires severe thermodynamic conditions, such as high temperatures (>100–250 °C), high pressure, or the presence of concentrated mineral acids/alkali catalysts. Our TFA dissolution was performed under relatively mild conditions (50 °C for 2h, then room temperature), which are thermodynamically insufficient to hydrolyze the highly recalcitrant poly (ethylene terephthalate) backbone.

3. Minor alterations to end-groups (end-capping)

While the main chain remains intact, in-depth nuclear magnetic resonance (NMR) investigations have revealed that TFA does induce minor, specific chemical alterations at the extremities of the polymer chains. Studies by Kenwright et al. [2] and Tu et al. [3] have elegantly demonstrated that TFA reacts with the terminal hydroxyl (-OH) end-groups of PET to form trifluoroacetate end-caps via a mild esterification reaction. However, Kenwright et al. explicitly concluded that while this end-group modification occurs, "there is no significant degradation of the polymer chains" during TFA exposure.

In summary, the TFA treatment could reduce crystallinity (physical change) and induces minor terminal end-capping (chemical change at the extremities), but it preserves the integrity of the main-chain ester bonds. The resulting particles remain authentically high-molecular-weight PET.

We also performed DSC analysis to determine the crystallinity of the original post-consumer PET materials and the TFA-prepared Pc-PET MPs. Crystallinity was measured and calculated according to Lu et.al [4]:

$$\%Crystallinity= \left( \frac{{\Delta H}_{m}-{\Delta H}_{cc}}{{\Delta H}_{m}^{0}} \right)\times100\%$$

where ${\Delta H}_{m}$ is the enthalpy of melting (J/g), ${\Delta H}_{cc}$ is the enthalpy of cold crystallization (J/g), and ${\Delta H}_{m}^{0}$ is the enthalpy of melting for a 100% crystalline PET sample, which is 140.1 J/g.

The DSC results of four post-consumer PET materials are shown as Figure S16, after mixing these materials at a 1:1:1:1 mass ratio and processing them by TFA dissolution/precipitation, the final crystallinity of raw materials and Pc-PET MPs are shown in Table S8.

These results confirm that TFA processing substantially reduces PET crystallinity. However, this does not alter the main conclusion of our study:

First, the TFA-prepared MPs were used as a standardized cellular exposure model because uniform particle size and stable suspension are required for reproducible cell imaging and cytotoxicity assays.

Second, all enzymes compared in the PET MPs degradation assays were tested using the same substrate preparation, so the reduced crystallinity would not selectively favor AhPETase^M1^ over the benchmark enzymes.

Third, the catalytic advantage of AhPETase was also established using untreated commercial semi-crystalline PET powder in our initial benchmark assays.

Thus, while we now explicitly acknowledge that TFA-prepared Pc-PET MPs are more accessible than highly crystalline environmental PET debris, the relative enzymatic superiority and the proof-of-concept cellular detoxification conclusion remain supported.

**DSC analysis for determining PET crystallinity**

To clarify the crystallinity of TFA treated PET materials, DSC was used to determine thermodynamic property. PET samples were first heated from 40 to 300 °C at 5 °C/min, held at 300 °C for 1 min, cooled from 300 to 30 °C at -5 °C/min and held at 30 °C for 1 min under nitrogen stream.

References for this note:

[1] Abedsoltan H. (2023) A focused review on recycling and hydrolysis techniques of polyethylene terephthalate. ***Polym. Eng. Sci.*** 63(9): 2651-2674.

[2] Kenwright A M. et al. (1999) End group modification in poly (ethylene terephthalate). ***Polymer*** 40(8): 2035-2040.

[3] Tu Y Y. et al. (2021) The effect of trifluoroacetic acid on molecular weight determination of polyesters: an in-situ NMR investigation. ***Chin. J. Polym. Sci.*** 39(12): 1590-1596.

[4] Lu, H., Diaz, D.J., Czarnecki, N.J. et al. Machine learning-aided engineering of hydrolases for PET depolymerization. ***Nature*** 604, 662–667 (2022).

**Supplementary Note 3 UPLC details for the products quantification criteria**

First, we would like to clarify that in our UPLC analysis, intermediate products, specifically bis(2-hydroxyethyl) terephthalate (BHET), were strictly monitored alongside TPA and MHET. However, BHET peaks were found to be negligible during the degradation process as Figure c showed below.

Secondly, and more importantly, quantifying the total release of TPA and MHET (the predominant, terminal soluble products) is the widely established and adopted method for evaluating enzymatic PET depolymerization activity in the field. This standardized quantification approach has been widely employed in several PETase studies, including FAST-PETase [1], HotPETase [2], DepoPETase [3], ThermoPETase [4], MG8 [5] et al. Because BHET is merely a transient intermediate, the cumulative concentration of MHET and TPA accurately captures >99% of the soluble monomeric products, thereby reflecting the true extent of polymer backbone cleavage.

Supplementary note 3 figure. UPLC details for the products quantification criteria. shows the UPLC results of standard TPA (Aladdin, P108506, purity ≥99%) and MHET (Aladdin, H302233, purity ≥98%). Figure (b) shows the UPLC results of BHET (Sigma-Aldrich, 165151). Figure (c) shows the UPLC results of AhPETase screening process.

References for this note:

[1] Lu, H. et al. (2022) Machine learning-aided engineering of hydrolases for PET depolymerization. ***Nature*** 604, 662-667. 10.1038/s41586-022-04599-z

[2] Bell, E.L. et al. (2022) Directed evolution of an efficient and thermostable PET depolymerase. ***Nat. Catal****.* 5, 673-681. 10.1038/s41929-022-00821-3

[3] Shi, L. et al. (2023) Complete Depolymerization of PET Wastes by an Evolved PET Hydrolase from Directed Evolution. ***Angew. Chem. Int. Ed*.** 62. 10.1002/anie.202218390

[4] Son, H.F. et al. (2019) Rational Protein Engineering of Thermo-Stable PETase from Ideonella sakaiensis for Highly Efficient PET Degradation. ***ACS Catal.*** 9, 3519-3526. 10.1021/acscatal.9b00568

[5] Eiamthong, B. et al. (2022) Discovery and Genetic Code Expansion of a Polyethylene Terephthalate (PET) Hydrolase from the Human Saliva Metagenome for the Degradation and Bio‐Functionalization of PET. ***Angew. Chem. Int. Ed*.** 61. 10.1002/anie.202203061

**Supplementary Note 4 Details of approaches, configuration, and iterations of the fine-tuning process.**

1. Dataset construction

As outlined in the Experimental Section, the dataset comprised positive and negative target sequences. The positive dataset was established by searching for "Poly (ethylene terephthalate) hydrolase" within the UniProtKB database (up to March 2024), yielding 927 valid sequences (14 reviewed in Swiss-Prot and 913 unreviewed in TrEMBL). The negative dataset was generated by randomly sampling from the entire Swiss-Prot database (excluding entries labeled with PET hydrolase activity). To ensure robust training, we applied an upsampling strategy with a negative-to-positive ratio of 10:1 (i.e., using 10 times the quantity of negative samples compared to positive samples).

2. Model Architecture and Hardware Configuration

The ESM-1b fine-tuning process was executed on 8 NVIDIA A100 GPUs at the Westlake University High-Performance Computing Center. As depicted in Figure 1a, a binary classification head was appended to the pre-trained ESM-1b model. The model was fine-tuned using a top-layer optimization strategy, leveraging the ESM-EZY pipeline previously developed by our group (<https://github.com/westlake-repl/ESM-Ezy>).

3. 5-Fold Cross-Validation and Iterations

To rigorously assess model robustness and prevent overfitting, we employed a standard 5-fold cross-validation strategy. During this stage, the positive dataset was randomly partitioned into five equal folds, while the negative dataset remained unchanged. In each of the five iterations, four folds were used as training positives, and the remaining one fold was held out as the test set. The model was fine-tuned across these five distinct fold settings in parallel. For each fold, training was run for a maximum of 30 epochs, and the optimal epoch was selected using an early stopping mechanism based on validation loss. Finally, the model's robustness was evaluated on the test sets using metrics including ROC-AUC, Accuracy, Precision, Recall, and F1-score, demonstrating consistently high performance as summarized in Table S1.

4. Hyperparameter Configurations

The specific hyperparameters employed during the iterations were as follows:

| Batch_size | Learning_rate | Epochs | Early_stop | Class_num |
| --- | --- | --- | --- | --- |
| 24 | 1.0 ×10 ^−6^ | 30 | 3 | 2 |

Where, early stop represents for patience parameter: training halts if metrics do not improve for 3 consecutive epochs); Class_num represents for binary classification: positive vs. negative.

**Supplementary Note 5 Strategy for normalizing the model to prevent overfitting.**

Preventing overfitting is indeed the paramount challenge when adapting massive pre-trained Protein Language Models like ESM-1b (which contains 650 million parameters) to a specific, relatively small dataset.

To ensure the model learns generalized evolutionary and structural features relevant to PET hydrolysis, rather than simply memorizing the training data, we implemented a comprehensive, multi-level anti-overfitting and normalization strategy:

1. Parameter-Efficient Partial Fine-Tuning

This was our primary defense against overfitting. Instead of updating all 650 million weights of the entire ESM-1b architecture (Full Fine-Tuning), we employed a highly constrained Partial Fine-Tuning strategy. Specifically, we completely froze the parameters of the lower 32 transformer layers of the ESM-1b base model, utilizing them strictly as a semantic feature extractor. We only unfroze and fine-tuned the uppermost transformer layer (Layer 33) alongside the newly initialized linear classification head. This drastic reduction in trainable parameters strictly constrains the mathematical capacity of the model to overfit, while allowing the top layer to adapt specifically to PETase features.

2. Rigorous Early Stopping Mechanism

During the training process, we continuously monitored the loss on the hold-out validation set. We implemented an Early Stopping algorithm with a strict patience parameter set to 3. This ensured that the training process halted immediately at the exact optimal epoch before the model could begin memorizing training noise.

3. Dynamic Negative Sampling

To further prevent the model from memorizing specific negative examples, we implemented a Dynamic Negative Sampling strategy to balance the large volume of negative data against the smaller positive sample set during the data loading phase [1, 2]. This means the model encounters diverse combinations of negative sequences across different training epochs, acting as a powerful implicit regularization technique that forces the model to learn true discriminative boundaries.

4. Conservative Optimization and 5-Fold Cross-Validation

We utilized the Adam optimizer with an exceptionally conservative learning rate of 1.0×10^−6^ to ensure smooth gradient updates. Finally, the entire process was enveloped in a strict 5-fold cross-validation framework. The consistently high evaluation metrics achieved across all unseen test folds empirically prove that our normalization and anti-overfitting strategies were highly successful (as shown in Table S1).

References related to this question:

[1] Zhang W. et al. (2013). Optimizing top-n collaborative filtering via dynamic negative item sampling. ***36^th^ ACM SIGIR IR*** 785-788.

[2] Qian, H. et al. (2025) ESM-Ezy: a deep learning strategy for the mining of novel multicopper oxidases with superior properties. ***Nat. Commun.*** 16, 3274

**Supplementary Note 6 Significance of the selected ML model for zero-shot protein fitness prediction.**

The significance of employing zero-shot machine learning models for protein fitness prediction lies in their ability to computationally evaluate the impact of every possible amino acid substitution across the entire sequence without requiring any task-specific, high-throughput experimental data (e.g., Deep Mutational Scanning). For a newly discovered, uncharacterized enzyme like AhPETase, this capability is revolutionary, as it drastically shrinks the vast mutational search space and accelerates rational enzyme engineering.

To achieve highly robust and unbiased predictions, we deliberately selected an ensemble of state-of-the-art predictors, which we theoretically categorized into two distinct paradigms to ensure comprehensive biological insight:

1. MSA-Based Models (EVE, Tranception, EVmutation, MSA Transformer)

These models exhibit ability in capturing co-evolutionary epistatic interactions. By analyzing Multiple Sequence Alignments (MSAs) of homologous proteins, these models explicitly model the statistical dependencies between specific residue pairs. For instance, EVmutation utilizes an unsupervised statistical method for predicting the effects of mutations [1], while EVE employs deep generative variational autoencoders (VAEs) [2]. Tranception and MSA Transformer leverage attention mechanisms over MSAs [3, 4]. The significance of these models is their unparalleled ability to deduce whether a mutation will disrupt critical, co-evolved interaction networks within the enzyme's architecture.

2. Evolutionary Language Models (ESM-1v, ESM-2, SaProt)

These models represent the forefront of Protein Language Models (PLMs). Unlike MSA-based methods, they do not rely on the availability or quality of homologous sequences. Instead, models like ESM-1v and ESM-2 (trained on up to billions of unaligned sequences) learn the profound, underlying "protein grammar" and global semantic context of amino acids directly from raw sequences [5, 6]. Crucially, we included SaProt, a structure-aware PLM that integrates 3D structural tokens [7]. The significance here is that SaProt bridges the gap between sequence and spatial conformation, evaluating whether a mutation induces favorable or detrimental steric clashes in 3D space.

3. The Power of Ensemble Strategy

The ultimate significance of our approach is the integration (Ensemble) of these diverse models. By calculating the normalized aggregated scores from both MSA-based (local co-evolutionary constraints) and Evolutionary-based (global deep semantics and 3D structural context) paradigms, we effectively eliminated the inherent biases or blind spots of any single predictor, which is also proposed and proved to be practical in recent research [8, 9].

References related to this question:

[1] Hopf T A. et al. (2017) Mutation effects predicted from sequence co-variation. ***Nat. Biotechnol.*** 35(2): 128-135.

[2] Frazer J. et al. (2021) Disease variant prediction with deep generative models of evolutionary data. ***Nature*** 599(7883): 91-95.

[3] Notin P. et al. (2022) Tranception: protein fitness prediction with autoregressive transformers and inference-time retrieval. ***ICML*** 16990-17017.

[4] Rao R M. et al. (2021) MSA transformer. ***ICML*** 8844-8856.

[5] Meier J. et al. (2021) Language models enable zero-shot prediction of the effects of mutations on protein function. ***Adv. Neural Inf. Process. Syst.*** 34: 29287-29303.

[6] Lin Z. et al. (2023) Evolutionary-scale prediction of atomic-level protein structure with a language model. ***Science*** 379(6637): 1123-1130.

[7] Su J. et al. (2024) SaProt: Protein Language Modeling with Structure-aware Vocabulary. ***ICLR***

[8] Tran V Q. et al. (2026) Rapid directed evolution guided by protein language models and epistatic interaction. ***Science*** eaea1820.

[9] Ding K. et al. (2024) Machine learning-guided co-optimization of fitness and diversity facilitates combinatorial library design in enzyme engineering. ***Nat. Commun.*** 15(1): 6392.

**Supplementary Note 7 Statistical analysis in Figure 4d, 4e and 4f.**

We confirm that comprehensive statistical analyses were performed using IBM SPSS Statistics 24 for the cell viability assays presented in Figure 4d, 4e and 4f. The data are expressed as the mean ± standard deviation (s.d.) from three independent biological replicates (n=3).

Prompted by your insightful comment, we have thoroughly re-audited our statistical workflow to explicitly document how the assumptions underlying our choice of tests (normality and homoscedasticity) were strictly met or appropriately addressed:

1. Assumption of Normality

Prior to performing mean comparisons, the normality of the data distribution for each experimental group was quantitatively assessed using the Shapiro-Wilk (S-W) test, which is robust for small sample sizes. The S-W test results confirmed that our data did not significantly deviate from a normal distribution (P>0.05) across the vast majority of evaluated groups). This assumption was further visually corroborated by the normal P-P plots, justifying the use of parametric tests.

2. Assumption of Variance Homoscedasticity and Test Selection

To assess the homogeneity of variances across the treatment groups, we utilized Levene’s test (shown in following table). Based on these results, our statistical approach was carefully tailored to the specific characteristics of each dataset:

**For datasets meeting the assumption of equal variances:** In the majority of our experimental datasets, Levene’s test confirmed homoscedasticity (P>0.05). For these groups, we appropriately applied the standard One-Way Analysis of Variance (ANOVA) followed by Tukey’s post hoc test for pairwise multiple comparisons.

**For the dataset violating the assumption of equal variances:** In Fig. 4d group, Levene’s test yielded a significant result (P=0.009 < 0.05), indicating heteroscedasticity. For this specific dataset, to ensure strict statistical validity and avoid Type I errors, we rigorously switched to the more robust Welch’s ANOVA, followed by Dunnett’s T3 post hoc test, which does not assume equal variances.

While the Shapiro-Wilk test indicated potential deviations from normality in certain subgroups, this is a known limitation of the test when dealing with small sample sizes (n=3) typical in in vitro cellular assays. In such cases, ANOVA (and Welch's ANOVA) remains highly robust to moderate violations of normality, provided that variances are accounted for. This tailored, assumption-driven statistical approach guarantees that the significance markers (∗P<0.05) reported in our figures are completely robust.

**Supplementary Note 8 Parameters and criteria used for the bioinformatics programs.**

1. Phylogenetic & Sequence Analysis: Tools for plasmids design, sequence alignment, tree construction, and signal peptide annotation (e.g., SnapGene, MEGA12, iTOL v6, ESPript 3.0, SignalP 6.0).

2. Structural Biology & Molecular Dynamics: Programs for 3D visualization, docking, and atomistic simulations (e.g., PyMOL, Boltz-2, GROMACS).

3. Machine Learning & Zero-Shot Predictors: The suite of state-of-the-art Protein Language Models and evolutionary models used for variant effect prediction (e.g., ESM-1v, ESM-2, SaProt, EVE, EVmutation, MSA Transformer, Tranception).

For complex computational pipelines, specifically the phylogenetic tree construction (MEGA 12), Molecular Dynamics (MD) simulations (GROMACS), molecular docking (Boltz-2) and ensemble zero-shot protein fitness prediction (ESM-1v, ESM-2, SaProt, EVE, EVmutation, MSA Transformer, Tranception), the detailed methodologies, tailored parameters, and rigorous quality control criteria (e.g., 5-fold cross-validation, 1000 bootstrap replicates for phylogeny, and specific force fields/equilibration steps for MD) have been thoroughly documented in the respective subsections of the revised Experimental Section.

For standard sequence analysis, visualization, and annotation tools (e.g., iTOL v6, SnapGene, SignalP, PyMOL, ESPript 3.0), we utilized the default parameters as recommended by the software developers, unless specifically noted otherwise in the text.
